# Early selection of *bZIP73* facilitated adaptation of *japonica* rice to cold climates

**DOI:** 10.1038/s41467-018-05753-w

**Published:** 2018-08-17

**Authors:** Citao Liu, Shujun Ou, Bigang Mao, Jiuyou Tang, Wei Wang, Hongru Wang, Shouyun Cao, Michael R. Schläppi, Bingran Zhao, Guoying Xiao, Xiping Wang, Chengcai Chu

**Affiliations:** 1 State Key Laboratory of Plant Genomics and National Centre for Plant Gene Research (Beijing), Institute of Genetics and Developmental Biology, Chinese Academy of Sciences, Beijing, 100101 China; 2 Department of Horticulture, Michigan State University, East Lansing, MI 48824 USA; 3 State Key Laboratory of Hybrid Rice, China National Hybrid Rice Research and Development Center, Changsha, 410125 China; 4 Department of Biological Sciences, Marquette University, Milwaukee, WI 53233 USA; 5 Institute of Subtropical Agriculture Chinese Academy of Sciences, Changsha, 410125 China; 6 Beijing Normal University, Beijing, 100875 China; 7 University of Chinese Academy of Sciences, Beijing, 100049 China

## Abstract

Cold stress is a major factor limiting production and geographic distribution of rice (*Oryza sativa*). Although the growth range of *japonica* subspecies has expanded northward compared to modern wild rice (*O. rufipogon*), the molecular basis of the adaptation remains unclear. Here we report *bZIP73*, a bZIP transcription factor-coding gene with only one functional polymorphism (+511 G>A) between the two subspecies *japonica* and *indica*, may have facilitated *japonica* adaptation to cold climates. We show the *japonica* version of bZIP73 (bZIP73^Jap^) interacts with bZIP71 and modulates ABA levels and ROS homeostasis. Evolutionary and population genetic analyses suggest *bZIP73* has undergone balancing selection; the *bZIP73*^*Jap*^ allele has firstly selected from standing variations in wild rice and likely facilitated cold climate adaptation during initial *japonica* domestication, while the *indica* allele *bZIP73*^*Ind*^ was subsequently selected for reasons that remain unclear. Our findings reveal early selection of *bZIP73*^*Jap*^ may have facilitated climate adaptation of primitive rice germplasms.

## Abstract (web summary)

*Japonica* rice can grow further north than wild or *indica* rice and is more tolerant of cold climates. Here, the authors show that *bZIP73* likely underwent selection in the early phase of rice domestication to facilitate cold tolerance in *japonica* by modulating ABA and ROS homeostasis.

## Introduction

Rice (*Oryza sativa*) is one of the most important cereal crops feeding more than half of the world’s population^1,2^. As a tropical plant, rice is more sensitive to cold stress than other cereal crops such as wheat (*Triticum aestivum*) and barley (*Hordeum vulgare*)^3,4^. Therefore, in high-latitude and high-altitude regions, cold stress is a major limiting factor for rice growth and production^3,5,6^. Asian rice consists of two geographically and genetically diverged subspecies, *indica* and *japonica*, which are distinct in their ability to tolerate cold stress^7–9^. In general, *japonica* cultivars are more cold tolerant than *indica* and predominate temperate zones (e.g., northern China, Korea, and Japan), whereas *indica* cultivars grow in tropical and subtropical regions (e.g., southern China and South Asia)^10^.

Despite the divergence of rice cultivars, the domestication history of cultivated rice is uncertain due to contrasting evidences. Garris et al.^11^ revealed the deep genetic structure and diversity of different rice ecotypes^11^, and, based on phylogenetic studies, Londo et al.^12^ and Civáň et al.^13^ proposed multiple independent domestication events for cultivated rice^12,13^. In contrast, Huang et al.^14^ and Choi et al.^15^ proposed that rice was initially domesticated as *japonica*, while subsequent backcrosses to different groups of common wild rice (*O. rufipogon*) generated *indica*, *aus*, and other subpopulations^14,15^. However, two recent studies using large-scale rice populations found limited introgression signals around key domestication genes^16,17^. Although the common wild rice is considered the direct ancestor of modern cultivated rice, the species itself has diverged into several groups that may represent different origins of modern cultivated rice^12,14,18,19^. Moreover, wild rice populations did not expand to eastern and northern China until the Last Glacial Maximum ~16,000 years ago when warm and wet conditions emerged^20^. During the expansion to cold climates, positive selections on standing variations of functional genes probably facilitated rice adaptation to cold stress^21^. For example, artificial selection of a single-nucleotide polymorphism (SNP) in *COLD1*, a gene regulating G-protein signaling, enhanced cold tolerance in *japonica* rice^21^; and the wild rice-derived haplotype of *CTB4a* was retained by artificial selection during *temperate japonica* domestication in cold habitats^22^.

Cold tolerance is a complex trait^3^. Several genes associated with cold stress have been identified so far, which can be divided into two groups. The first group consists of functional components that directly protect plant cells from cold stress, i.e., enzymes in metabolic pathways^23^. The second group consists of genes that regulate gene expression during stress responses, i.e., signaling components and transcription factors (TFs)^23^. Together with their target genes, TFs can constitute regulons that participate in signal transduction for activating or repressing genes involved in cold stress responses. Thus, TFs are excellent candidates for modification of complex traits in crops^24^.

The basic leucine zipper (bZIP) family is one of the most diverse TF families in vascular plants and has 91 members in rice^25,26^, some of which are involved in cold stress responses^25,27–30^. For example, OsbZIP52/RISBZ5 functions as a negative regulator of cold stress tolerance^31^; OsbZIP38/LIP19 interacts with OsbZIP87/OBF1 and plays important roles in cold signaling pathways^32^; and a number of bZIP genes are up- or down-regulated under cold stress conditions^25,33^. However, a potential contribution of bZIP TFs to the northward expansion of *japonica* has not yet been investigated. To fill this gap, we combined association studies, population genetics, and stress-response profiling for the identification of candidate *bZIP* genes. Our results show that *bZIP73* contributes to low-temperature seedling survivability (LTSS) and may have been a target of selection during the domestication of *japonica* rice. Molecular and transgenic studies revealed that the *japonica* version of bZIP73 (bZIP73^Jap^) interacts with bZIP71 and functions by modulating abscisic acid (ABA) level and reactive oxygen species (ROS) homeostasis, which could significantly enhance rice tolerance to cold. Evolutionary and population genetic analyses suggest that the two alleles of *bZIP73* have undergone several rounds of artificial selection in both common wild rice and cultivated rice, indicating that balancing selection may have occurred on this gene. Selection of *bZIP73*^*Jap*^ in the direct ancestor of *japonica* suggests that the gene may have contributed to cold climate adaptation during the early stages of *japonica* domestication.

## Results

### *bZIP73* is an excellent cold tolerance candidate gene in rice

To systematically study natural variations of *bZIP* genes, we combined two published *Oryza* resequencing populations, the USDA (United States Department of Agriculture) rice mini-core population^34^ and a large collection of common wild rice^14^. The combined population has a total of 641 cultivars or accessions collected from over 15 geographic regions (Supplementary Fig. 1)^35^, which includes 66 *indica*, 57 *japonica*, 81 other cultivars (*aus*, *aromatic*, and admixture varieties), and 435 common wild rice strains. From the high-density SNP set (2.3 M) we previously generated for the combined population^34^, a total of 27,008 SNPs including 1010 non-synonymous substitutions were identified in the coding region of 91 bZIP genes. We measured the LTSS of the mini-core population for association analyses with the *bZIP* gene family^36^. Unlinked amino-acid (aa) variations were used as association markers. After correcting for population structure and adjusting the false discovery rate (FDR) for multiple testing^37^, the following 8 out of 91 *bZIP* genes were significantly correlated with the LTSS phenotype (*p* < 0.05, FDR_BH) (Supplementary Table 1): *OsbZIP08*, *OsbZIP35*, *OsbZIP38*, *OsbZIP46*, *OsbZIP63*, *OsbZIP72*, *OsbZIP73*, and *OsbZIP76*.

The northward expansion of *japonica* was probably achieved by local adaptation to cold stress. We thus scanned for genomic footprints of local adaptation using a linkage disequilibrium (LD)-based *ω* statistics^38^. Of the eight LTSS-correlated *bZIP* genes, only *bZIP73* showed excessive LD increments, indicating that a positive selection for *bZIP73* occurred in *japonica* (Supplementary Table 2). These results strongly suggest that *bZIP73* is an excellent candidate to investigate the *japonica* adaptation to a cold climate.

### The TF bZIP73 is diverged between *japonica* and *indica*

*bZIP73* (*LOC_Os09g29820*) encodes a typical bZIP protein (173 aa) that contains a single SNP affecting the C terminus of the protein sequence (Supplementary Fig. 2), which is diverged between *japonica* and *indica* (+511 bp, G>A; +171 aa, Glu>Lys; the first bp or the first aa of the coding region was defined as +1) (Supplementary Table 3 and Supplementary Data 4). We hereafter used the names of *bZIP73*^*Jap*^ and *bZIP73*^*Ind*^ for the *japonica* and *indica* haplotypes, respectively.

Using bZIP73::GFP fusion proteins in rice protoplasts, we found that both bZIP73 versions exclusively co-localized with a nuclear marker, OsMADS3::mCherry (Supplementary Fig. 3a, b). Truncated bZIP73 proteins (binding domain (BD)-73N2, 1–156 aa; BD-73M, 77–156 aa; BD-73^Jap/Ind^C2, 77–173 aa), which contained both the basic region and leucine zipper domains, had transactivation activities in yeast (Supplementary Fig. 3c, d). However, neither the full-length CDS (BD-73^Jap/Ind^, 1–173 aa) nor smaller truncated constructs (BD-73N1, 1–99 aa, containing the basic region domain; BD-73^Jap/Ind^C1, 100–173 aa, containing the leucine zipper domain) of bZIP73 had transactivation activities in yeast (Supplementary Fig. 3c, d). These results indicate that the bZIP domain (basic region and leucine zipper) of bZIP73 is required for its transactivation activity, while the N-terminal (1–76 aa) and the C-terminal (157–173 aa) domains may be negative regulators of the transcriptional activity of bZIP73.

### A SNP in *bZIP73* alters cold sensitivity between *japonica* and *indica* subgroups

To determine whether *bZIP73* was under environmental control, we analyzed its expression profile under different abiotic stresses. Transcript levels of *bZIP73* increased after 1 to 6 h of cold, ABA, and H_2_O_2_ treatments in roots and/or shoots of rice seedlings (Supplementary Fig. 4), indicating that *bZIP73* may function in ABA-dependent cold signaling pathways.

**Fig. 1:**
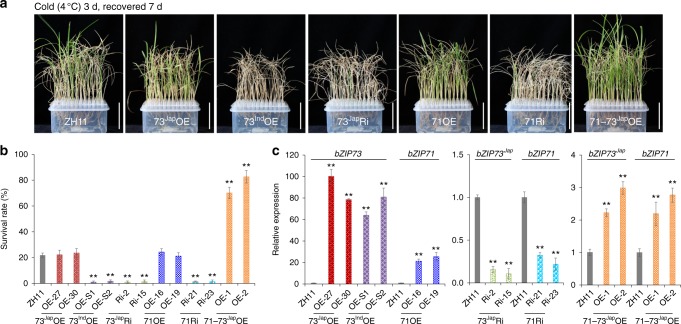
Assessment of cold stress tolerance of *bZIP73* and *bZIP71* transgenic rice. **a** Overexpression lines *bZIP73*^*Jap*^ (73^Jap^OE), *bZIP73*^*Ind*^ (73^Ind^OE), and *bZIP71* (71OE), co-expression lines *bZIP71*-*bZIP73*^*Jap*^ (71–73^Jap^OE), and RNAi lines *bZIP73*^*Jap*^ (73^Jap^Ri) and *bZIP71* (71Ri) were cold treated at 4 °C for 3 days and are shown after recovery at 28 °C for 7 days. ZH11, wild-type Zhonghua 11. **b** Survival rates of ZH11 and transgenic lines recovered after cold treatment. Scale bars, 5 cm. **c** Relative expression levels of *bZIP73* and *bZIP71* in ZH11 and transgenic lines measured by real-time PCR under normal condition. Target genes for measurement are indicated on top of the plot, and expression levels in ZH11 (wild-type plants) was used as reference (1×) to show expression fold change (*y*-axes) in transgenic lines as indicated on *x*-axes. Due to the different scale of relative expression levels, data were separated into three subplots. All data were collected using two independent homozygous lines indicated on *x*-axes with three biological replicates (*n* = 288 each). Error bar, standard deviation; **p < 0.01, two-tailed *t*-test in comparison to ZH11

To further characterize the function of *bZIP73*, we overexpressed *bZIP73*^*Jap*^ (73^Jap^OE) and *bZIP73*^*Ind*^ (73^Ind^OE) separately in the *japonica* cultivar Zhonghua 11 (ZH11) and silenced *bZIP73*^*Jap*^ (73^Jap^Ri) using RNA interference (RNAi) in ZH11. Two-week-old rice seedlings of homozygous transgenic lines (T_3_ generation) were exposed to cold stress (4 °C) for 3 days and subsequently returned to normal temperature (28 °C) for recovery (Supplementary Fig. 5). Our results showed that both 73^Ind^OE and 73^Jap^Ri lines were more sensitive to cold stress than the wild type (WT), because they had <2% survival rates (the percentage of recovered seedlings), which was significantly lower than the 22% survival rate of WT plants (Fig. 1a, b) (*p* < 0.01, two-tailed *t-*test). However, the survival rate of 73^Jap^OE lines was not significantly different from that of WT plants (Fig. 1a, b), indicating that the function of bZIP73 may require cofactors.

### bZIP73 interacts with bZIP71 and enhances cold tolerance

**Fig. 2:**
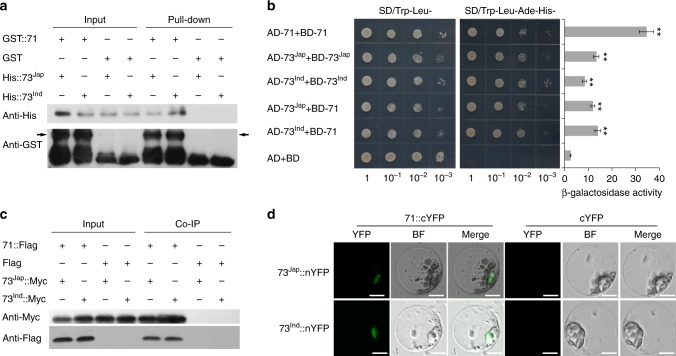
In vivo and in vitro interaction assays between bZIP71 and bZIP73. **a** In vitro pull-down assay. Left panel (Input), 10% of input before precipitation; Right panel (Pull-down), affinity purification using glutathione *S*-transferase (GST) or GST::71 as bait, and polyhistidine (His)::73^Jap^ or His::73^Ind^ as prey. Anti-His and anti-GST antibodies were used to detect His and GST peptides, respectively. Black arrows indicate anti-GST detection of GST::71, and + and − indicate that ingredients shown at the left were added or not added, respectively, to the experiment. **b** In vivo yeast two-hybrid assay. Sequences of bZIP73^Jap^, bZIP73^Ind^, and bZIP71 were fused to both the pGADT7 activation domain (AD, prey) and the pGBKT7 binding domain (BD, bait), and then transformed into yeast. Shown are growth phenotypes of yeast transformants on selective media of SD/Trp-Leu- (left panel) and SD/Trp-Leu-His-Ade- (central panel, interaction). Physical protein–protein interactions were revealed by yeast colony sizes and quantified by β-galactosidase activity (right panel) using a liquid culture assay with three biological replicates. Error bar, standard deviation; **p < 0.01, two-tailed *t*-test in comparison to the negative control, AD (pGADT7)+BD (pGBKT7). **c** In vivo co-immunoprecipitation (Co-IP) assay. Sequences of bZIP73^Jap^ and bZIP73^Ind^ were fused with a Myc tag, while bZIP71 were fused with a Flag tag. Vectors of 73^Jap^::Myc and 73^Ind^::Myc were transformed into transgenic rice lines expressing 71::Flag. Left panel (Input), before precipitation; Right panel (Co-IP), Anti-Myc and anti-Flag antibodies were used to detect Myc and Flag peptides, respectively. **d** In vivo bimolecular fluorescence complementation assay. bZIP73^Jap^ and bZIP73^Ind^ were both fused with the N-terminal half of yellow fluorescent protein (nYFP), while bZIP71 was fused with the C-terminal half of YFP (cYFP). BF, bright field. Merge is overlay of YFP and bright field images showing nuclear localization. Right panel, negative control. Scale bars, 10 µm

To identify potential bZIP73 cofactors, we performed a yeast two-hybrid (Y2H) screening by using full-length CDS of *bZIP73*^*Jap*^ fused to pGBKT7 (BD-73^Jap^) as bait, and identified 23 potential cofactors including another bZIP protein, bZIP71 (Supplementary Data 1). The interaction between bZIP71 and bZIP73^Jap/Ind^ was further confirmed through additional Y2H assays in vitro (Fig. 2b) and in vivo using bimolecular fluorescence complementation (BiFC) assays in rice protoplasts (Fig. 2d). Furthermore, we performed co-immunoprecipitation (Co-IP) experiments and found that both bZIP73^Jap^ and bZIP73^Ind^ were co-immunoprecipitated with bZIP71 in vivo (Fig. 2c). Consistent with these results, an in vitro pull-down assay also showed a direct physical interaction between bZIP73^Jap/Ind^ and bZIP71 (Fig. 2a). Together, these in vitro and in vivo experiments suggest that both bZIP73^Jap^ and bZIP73^Ind^ can interact with bZIP71.

Similar to other known bZIP interactions^32,39^, we hypothesized that the bZIP73-bZIP71 interaction may facilitate activation of bZIP73 through a similar mechanism as previously reported for OsbZIP38-OsbZIP87, which confers a cold signal switch in rice^32^. To test this, we co-overexpressed *bZIP73*^*Jap*^ and *bZIP71* in ZH11 (71–73^Jap^OE) and subjected seedlings to cold stress at 4 ℃ for 3 days (Supplementary Fig. 5). In agreement with our hypothesis, the seedling survival rate was positively correlated with expression levels of *bZIP71* and *bZIP73*^*Jap*^ (Fig. 1b, c), which was greatly improved to >70%, while that of wild-type ZH11 was only 22% (Fig. 1a, b). These results indicated that co-overexpression of *bZIP71* and *bZIP73*^*Jap*^ significantly improves rice tolerance to cold stress (*p* < 0.01, two-tailed *t-*test).

### bZIP71 enhances bZIP73^Jap^ activity to reduce ROS levels

**Fig. 3:**
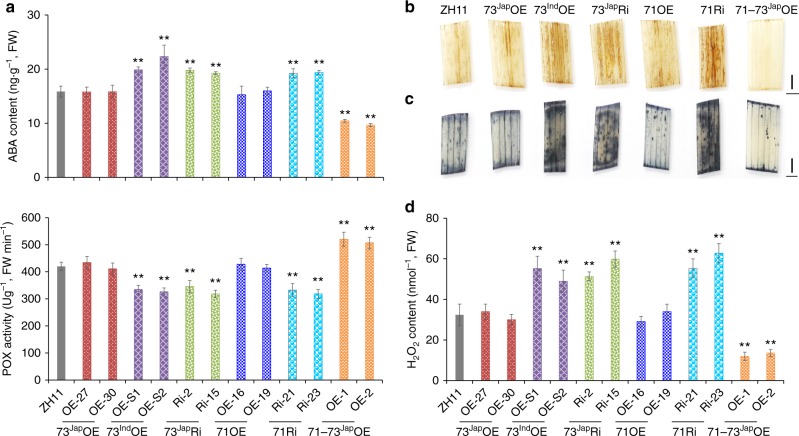
Metabolic responses of wild-type and transgenic rice lines under cold stress. **a** ABA content (*n* = 3, 32 plants/replication) and peroxidase (POX) enzyme activity (*n* = 8, 10 plants/replication) of transgenic lines under cold stress. **b** The 3,3’-diaminobenzidine (DAB) and **c** nitroblue tetrazolium (NBT) staining for the visualization of H_2_O_2_ and O_2_^−^, respectively, in cold-treated rice leaves. Scale bars, 2 mm. **d** H_2_O_2_ content (*n* = 8, 10 plants/replication) of transgenic lines under cold stress. OE, overexpression; Ri, RNA interference; 73^Jap^, *bZIP73*^*Jap*^; 73^Ind^, *bZIP73*^*Ind*^; 71, *bZIP71*; 71–73^Jap^OE, overexpression of both *bZIP71* and *bZIP73*^*Jap*^; FW, fresh weight. All experiments were conducted using two-week-old seedlings. Error bar, standard deviation; **p < 0.01, two-tailed *t*-test

To further understand how bZIP73^Jap^ regulates cold stress tolerance, we used chromatin immunoprecipitation (ChIP) sequencing to pull-down bZIP73^Jap^ targets and identified 669 non-TE genes that were precipitated with bZIP73^Jap^::Flag (Supplementary Data 2). These genes were enriched in Gene Ontologies (GOs) of abiotic stress responses (Supplementary Fig. 6), including four peroxidase (POX) precursor genes (*LOC_Os01g22249*, *LOC_Os03g02920*, *LOC_Os03g32050*, and *LOC_Os04g59210*) that share a typical regulatory G-box motif targeted by bZIP TFs in their promoters (Supplementary Data 3). The interaction between bZIP73^Jap^::Flag and the promoters of these four genes were further confirmed by ChIP quantitative PCR (qPCR) (Supplementary Fig. 7b). Transient expression assays in rice protoplasts showed that the promoter activities of these POX precursors were induced by cold treatment and enhanced by the addition of bZIP73^Jap^ (*p* *<* 0.01, two-tailed *t-*test) (Supplementary Fig. 8). Excitingly, the addition of bZIP71 significantly enhanced the transcription of these POX precursors under both normal condition and cold condition (*p* < 0.01, two-tailed *t-*test) (Supplementary Fig. 8). POXs are involved in defense against abiotic stresses through scavenging of ROS^40,41^. We found that both the expression of POX precursor genes and the POX activity were significantly up-regulated in 71–73^Jap^OE lines after cold stress (4 °C, 3 h) (Fig. 3a and Supplementary Fig. 9f) (*p* < 0.01, two-tailed *t-*test), but down-regulated in 73^Ind^OE, 73^Jap^Ri, and 71Ri lines (Fig. 3a and Supplementary Fig. 9b, d, e) (*p* < 0.01, two-tailed *t-*test). The cellular levels of O_2_^−^ and H_2_O_2_ were similar to each other in 73^Ind^OE, 73^Jap^Ri, and 71Ri lines, and were significantly higher compared to WT controls (Fig. 3b–d), indicating that these lines experienced more severe cold damage than WT plants (*p* < 0.01, two-tailed *t-*test). In contrast, cold damage in leaves of the 71–73^Jap^OE lines was significantly milder than in WT plants (*p* < 0.01, two-tailed *t-*test) (Fig. 3b–d).

### bZIP71 enhances bZIP73^Jap^ activity to decrease ABA levels

ABA plays a critical role in mediating cold stress responses in plants^42^. Under cold stress, increased levels of ABA may help improve cold tolerance of plants. However, since growth and stress responses are two cornerstones of the overall response of plants to the environment^43^, plants under continuous cold stress need to maintain a balance between cold stress adaptation and normal growth, and thus have evolved other mechanisms to avoid over-amplification of the signaling^43–45^. Previous studies indicated that sustained low levels of endogenous ABA could increase seedling tolerance to cold stress^46–48^. To test a potential connection between bZIP73 and ABA, we determined that ABA up-regulated *bZIP73*^*Jap*^ transcription (Supplementary Fig. 4), that 73^Jap^Ri lines had ABA hypersensitive phenotypes, and that 73^Jap^OE lines were neither ABA hypersensitive nor insensitive, while 71–73^Jap^OE lines were less sensitive to ABA at the early seedling stage (Supplementary Fig. 10). By analyzing promoters of ABA synthesis genes, we identified the two *Nine-Cis-Epoxycarotenoid Dioxygenase* (*NCED*) ABA synthesis genes *OsNCED3* and *OsNCED5* as having a G-box motif in their promoters (Supplementary Data 3). The in vitro and in vivo interactions between bZIP73^Jap^ and the promoters of these two genes were verified using ChIP-qPCR and electrophoretic mobility shift assays (EMSA) (Supplementary Fig. 7). Furthermore, we found that bZIP71 promotes such interactions in vitro (Supplementary Fig. 7a). Transient expression assays in rice protoplasts showed that bZIP73^Jap^ negatively regulates the expression of *OsNCED3* and *OsNCED5* under cold stress (4 °C, 3 h) (Supplementary Fig. 8), and the addition of bZIP71 enhanced the down-regulation (Supplementary Fig. 8). Therefore, compared to WT plants, expression levels of *OsNCED3* and *OsNCED5* and resulting ABA contents in 73^Ind^OE, 73^Jap^Ri, and 71Ri lines were significantly higher in 2-week-old seedlings after cold treatment (4 °C, 3 h) (Fig. 3a and Supplementary Fig. 9b, d, e) (*p* < 0.01, two-tailed *t-*test), while those in 71–73^Jap^OE lines were significantly lower (Fig. 3a and Supplementary Fig. 9f) (*p* < 0.01, two-tailed *t-*test). Taken together, these results indicated that bZIP73^Jap^ mediates cold tolerance by fine-tuning ABA levels, and this activity is enhanced by bZIP71.

### *bZIP73*^*Ind*^ introgression affects *japonica* cold tolerance

**Fig. 4:**
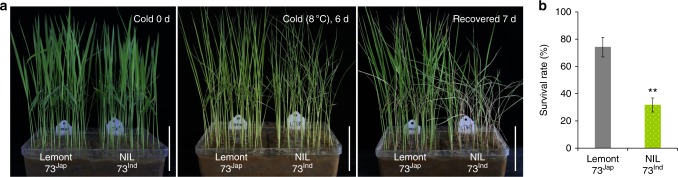
Increased sensitivity of *bZIP73*^*Ind*^ near-isogenic lines to cold stress. **a** Cold treatment of *bZIP73*^*Ind*^ near-isogenic lines (NIL) and Lemont (*japonica*) at 8 °C for 6 days and recovery (Recovered) at 28 °C for 7 days. NIL is introgression of *bZIP73*^*Ind*^ into the *japonica* variety Lemont. **b** Survival rates of Lemont and NIL after recovery from cold treatment. All data were collected with three biological replicates (*n* = 120 each). Error bar, standard deviation. Scale bars, 5 cm; **p < 0.01, two-tailed *t*-test in comparisons to the control (Lemont)

To validate the potential contribution of *bZIP73*^*Jap*^ to cold tolerance in different varieties, we generated a Lemont-background (*japonica*) near-isogenic line (NIL) carrying the *bZIP73*^*Ind*^ allele. The *bZIP73*^*Ind*^-NIL had a significantly lower survival rate (31.7%) than wild-type Lemont (74.2%) after cold treatment (Fig. 4) (*p* < 0.01, two-tailed *t-*test), demonstrating that *bZIP73*^*Jap*^ may be a promising cold tolerance candidate gene for crop breeding.

### *bZIP73* has undergone multiple rounds of positive selection

**Fig. 5:**
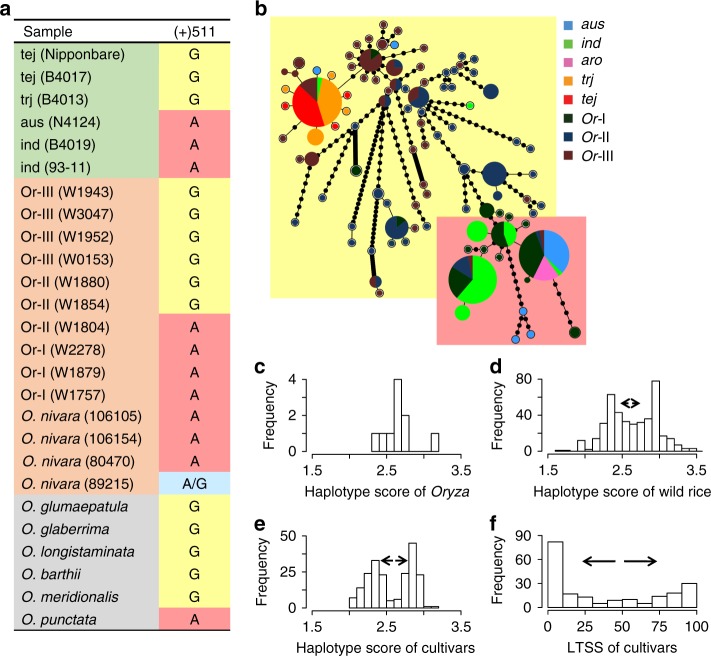
Phylogenetic analysis of the *bZIP73* gene. **a** Multiple DNA sequence alignments of 26 *OsbZIP73* genes. Nucleotide sequences of the FNP (+511 nt; the first nt of the coding region was defined as +1) are shown. Samples in the green background are cultivated rice; brown background indicates common wild rice *O*. *rufipogon* and *O. nivara*; gray background indicates other wild rice relatives. Tej, *temperate japonica*; trj, *tropical japonica*, ind, *indica*; aro, *aromatic*; Or, *O. rufipogon*. **b** Phylogenetic network of *bZIP73* genes with yellow and pink backgrounds denoting haplotypes carrying the *bZIP73*^*Jap*^ and *bZIP73*^*Ind*^ genotypes, respectively. The size of each node is proportional to the sample size. **c**–**e** Haplotype score distributions of AA-genome wild rice (**c**), *O. rufipogon* (**d**), and cultivated rice (**e**). **f** Phenotypic distribution of low-temperature seedling survivability (LTSS) of cultivated rice accessions. Arrows indicate the trend of genotypic or phenotypic differentiation

Our data suggest that *bZIP73* has undergone directional selection in *japonica* (Supplementary Table 2). To further investigate the evolutionary history of *bZIP73*, we collected a total of 26 *bZIP73* sequences from the *Oryza* genus. Multiple sequence alignment revealed that the functional polymorphism (FNP, +511) of *bZIP73* was differentiated in the rice progenitor *O. rufipogon* (Fig. 5a, Supplementary Table 3). All AA-genome wild rice relatives and 97% of *O. rufipogon* III (Or-III, the progenitor of *temperate japonica*^14^) examined contained the *japonica*-type *bZIP73* allele, indicating that *bZIP73*^*Jap*^ is the ancestral state, while 84% of *O. rufipogon* I (Or-I, the progenitor of *indica*^14^) contained the *indica*-type allele as the derived state (Fig. 5a, Supplementary Table 3 and Supplementary Data 4). As shown by zero heterozygosity and the observed allele frequency of the FNP, the two haplotypes of *bZIP73* are nearly fixed in the respective populations of cultivated and common wild rice (Supplementary Table 3 and Supplementary Data 4).

Local reduction of nucleotide diversity (*π*) indicates positive selection. We estimated the nucleotide diversity across a 20 kb genomic region flanking *bZIP73* in all rice subpopulations. The nucleotide diversity of *bZIP73* was very low in both cultivated (*π* < 0.0002) and wild rice (*π* < 0.001) populations compared to their genomic backgrounds (~0.002 and ~0.003, respectively^14^) (Supplementary Table 4). Moreover, the diversity of *bZIP73* sequences in *O. rufipogon* III and *indica* was significantly lower than that of their flanking regions (least significant difference (LSD), *α* = 0.05, Supplementary Table 4), indicating positive selection^49,50^. Extended low diversity in flanking regions of *japonica bZIP73* indicates a severe bottleneck during the selection of *bZIP73*^*Jap*^ (Supplementary Table 4), which might have been completed in *O. rufipogon* III and was subsequently passed down to *japonica*.

### *bZIP73*^*Jap*^ may have facilitated expansion of *japonica* rice

Phylogenetic analysis of *bZIP73* suggests that *bZIP73*^*Jap*^ was derived from *O. rufipogon* III, while *bZIP73*^*Ind*^ was derived from *O. rufipogon* I (Supplementary Fig. 11). The contemporary wild rice may not represent ancestral wild rice before domestication, because it has experienced extensive gene flow from cultivated rice due to open pollination^18^. Moreover, due to the lack of sequence diversity (Supplementary Table 4), the phylogeny of *bZIP73* fails to reveal binary relationships between rice populations (Supplementary Fig. 11). To resolve the reticulated relationships between *bZIP73* haplotypes, we constructed a phylogenetic network of *bZIP73*. A minimum spanning network (MSN) constructed from 98 haplotypes showed that the two alleles of *bZIP73* were derived from distinct common wild rice ecotypes (Fig. 5b). Similar to what we observed from the nucleotide diversity (Supplementary Table 4), the *bZIP73* MSN also showed that directional selection of *bZIP73*^*Jap*^ has undergone a more severe bottleneck (fewer steps from prototype to modern haplotype transition), while the *bZIP73*^*Ind*^ haplotype spread gradually with possible gene flow from its direct progenitor *O. rufipogon* I (Fig. 5b). Taken together with allele frequency (Supplementary Table 3) and phylogeny (Supplementary Fig. 11), we conclude parsimoniously that the two alleles of *bZIP73* distributed in *indica* and *japonica* were derived from different populations of ancestral wild rice.

**Fig. 6:**
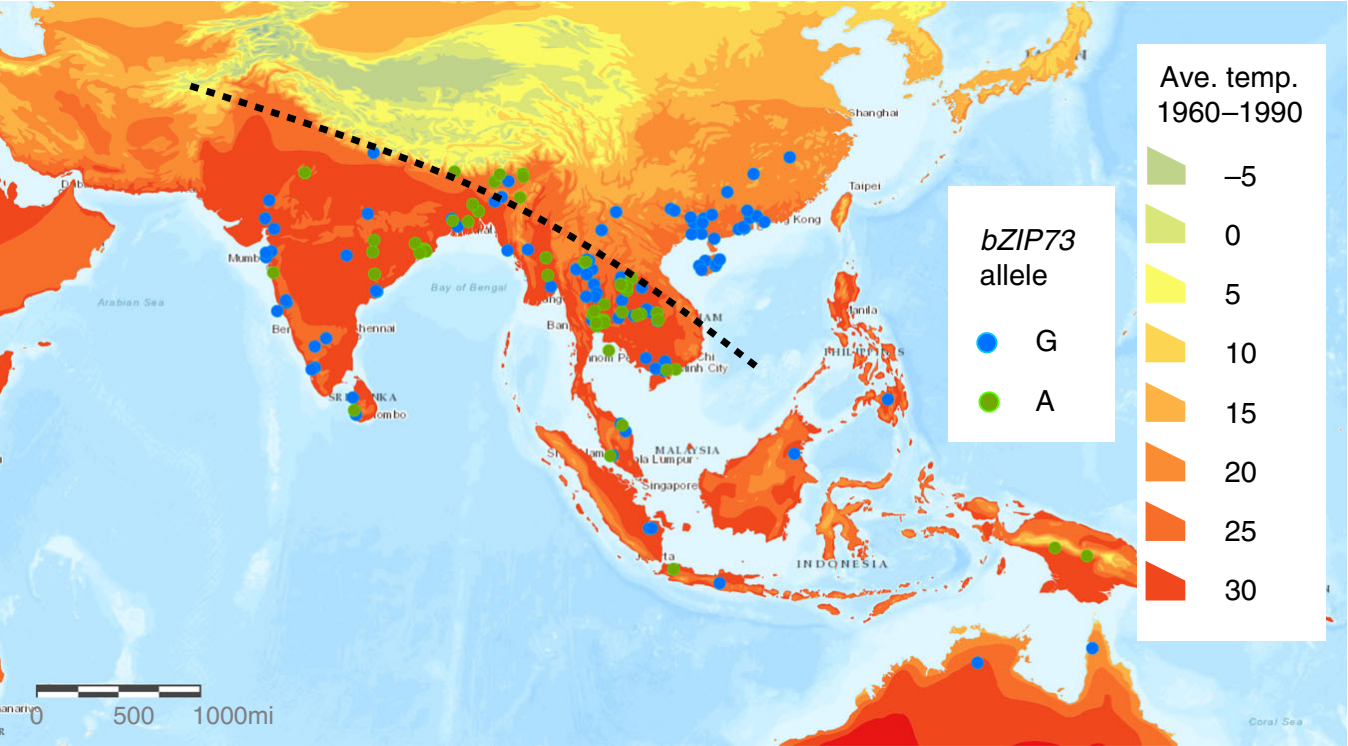
Geographic distribution of *bZIP73*^*Jap*/*Ind*^ in *O*. *rufipogon*. The *bZIP73*^*Jap*^ (G) and *bZIP73*^*Ind*^ (A) alleles are labeled in blue and green dots, respectively. Each dot represents the geographic origin of a common wild rice sample. The major geographic distribution of the *bZIP73*^*Ind*^ (A) allele is separated by a black dotted line. Terrestrial surface temperatures averaged from 1960 to 1990 A.D. are shown as a heat map with scale bars in °C. Copyright © Esri. All rights reserved

We further mapped the distribution of *bZIP73* alleles in our common wild rice samples to environmental factors such as terrestrial temperature. The *bZIP73*^*Ind*^ allele is predominantly distributed in India, Bangladesh, and the west of the Indo-China Peninsula, while the *bZIP73*^*Jap*^ allele is predominant in southern China (Fig. 6). Based on our functional studies in cultivated rice, the function of bZIP73 may have also been differentiated in the rice progenitor *O. rufipogon*. In addition, the average terrestrial temperature in southern China is at least 5 °C lower than that in the geographic distribution areas of *bZIP73*^*Ind*^ (Fig. 6), suggesting that the *bZIP73*^*Jap*^ allele may have helped the expansion of cultivated rice progenitors to cooler regions. Strikingly, based on phylogeographic analyses, Huang et al.^14^ reported that the common wild rice (Or-III) located in southern China was likely the direct ancestor of *japonica*^14^, all of which contain the *bZIP73*^*Jap*^ allele as shown in our data (Fig. 6). Taken together, the function of the *bZIP73*^*Jap*^ allele may have facilitated the northward expansion of Or-III-like common wild rice to southern China, and may have established a primitive germplasm for the domestication of *japonica* and its further northward expansion.

### *bZIP73* may have undergone balancing selection

**Fig. 7:**
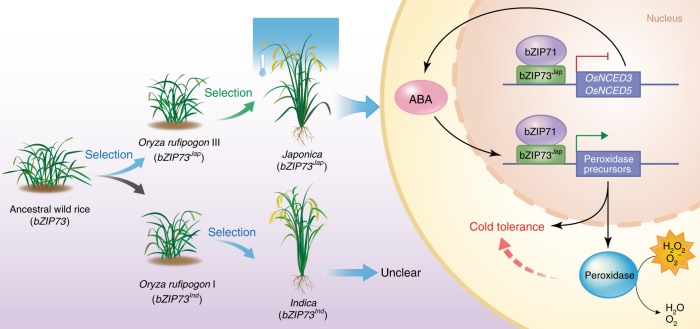
Step-wise selection model of *bZIP73* and the molecular mechanism of bZIP73^Jap^-orchestrated cold tolerance in *japonica* rice

Our data suggest that the *indica* genotype of *bZIP73* probably underwent positive selection, because *bZIP73*^*Ind*^ is the derived allele (Fig. 5a, b) that is almost fixed in *indica* (Supplementary Table 3 and Supplementary Data 4), and nucleotide diversity of *bZIP73*^*Ind*^ became locally reduced (Supplementary Table 4). Since *bZIP73*^*Jap*^ was selected at the early stage of rice domestication in *O. rufipogon* III (Supplementary Table 4) and further selected in *japonica* (Supplementary Table 2), selection of both *bZIP73* genotypes suggests that the gene has undergone balancing selection (Fig. 7). Based on the observed lack of heterozygosity (Supplementary Data 4) and the mode of spatial distribution of the two alleles (Fig. 6), this might have been a location-dependent balancing selection. To further test this hypothesis, we visualized the distribution of haplotype scores estimated from each sample in different populations. A unimodal distribution was observed in AA-genome wild rice (Fig. 5c), while a bimodal distribution was found for the *O. rufipogon* population and further enlarged in the cultivated rice population (Fig. 5d, e), which was also shown by the phenotypic (LTSS) distribution of cultivated rice (Fig. 5f). Together, these results suggest that artificial selection of *bZIP73* was initially started in *O. rufipogon* III and further fixed in *japonica*, which may have facilitated the northward expansion of Asian rice, while a second selection event for the *indica* genotype of *bZIP73* occurred later during the balancing selection of *bZIP73* (Fig. 7).

## Discussion

Cold tolerance is an ecologically significant trait for the adaptation of plants to cold climates. In this study, we conducted an association analysis of genomic resequencing data of 91 *bZIP* genes with LTSS phenotypic data using 203 *O*. *sativa* accessions from the USDA rice mini-core collection. By combining it with selective sweep scanning analyses, we found that *bZIP73* was the only *bZIP* gene associated with cold tolerance at the seedling stage that was a target for artificial selection during *japonica* domestication. Consistently, a *bZIP73*^*Ind*^ NIL in the *japonica* background was sensitive to cold stress compared to its recurrent *japonica* parent Lemont (Fig. 4). Moreover, *bZIP73*^*Jap*^ RNAi lines enhanced cold sensitivity of *japonica* rice (Fig. 1). These results suggest that *bZIP73*^*Jap*^ is potentially a valuable locus for breeding rice cultivars with superior tolerance to cold stress.

Most TFs do not function alone, but rather recruit intermediary proteins (cofactors) for effective initiation of transcription^39^. For example, DCA1 acts as a transcriptional co-activator of DST (drought and salt tolerance) and contributes to drought and salt tolerance in rice^39^, while OsbZIP38/LIP19 interacts with OsbZIP87/OBF1 and functions as a cold signal switch in rice^32^. In our study, neither individual 73^Jap^OE nor 71OE rice lines had altered cold tolerance, while either *bZIP73*^*Jap*^ or *bZIP71* RNAi lines were sensitive to cold stress (Fig. 1). We show here that bZIP73 physically interacts with bZIP71 both in vitro and in vivo (Fig. 2), and transgenic plants co-expressing *bZIP73*^*Jap*^ and *bZIP71* (71–73^Jap^OE) are significantly more cold tolerant than WT plants (Fig. 1). These results indicate that co-activation of bZIP71-bZIP73^Jap^ contributes to cold tolerance in rice.

bZIP73^Jap^ *trans*-activates POX gene precursors under both normal and cold conditions, which can be further enhanced by bZIP71 (Supplementary Fig. 8). This enhanced POX activity (Fig. 3a), together with decreased ROS levels by scavenging H_2_O_2_ and O_2_^−^ molecules (Fig. 3b-d), results in improved cold tolerance of transgenic 71–73^Jap^OE lines (Fig. 1). In contrast, transgenic lines overexpressing *bZIP73*^*Ind*^ in a *japonica* background are sensitive to cold stress (Fig. 1). This can be explained with a co-dominant-negative scenario in which both bZIP73^Ind^ and endogenous bZIP73^Jap^ competitively bind to bZIP71 (Supplementary Fig. 12), thus reducing the amount of endogenous bZIP73^Jap^-bZIP71 complex and leading to reduced POX activity and increased ROS levels (Fig. 3), ultimately causing cold sensitivity of 73^Ind^OE lines. In agreement with this, it was previously shown that *COLD1*^*Ind*^ OE lines were less cold tolerant than WT plants as a consequence of competitive binding of COLD1^Ind^ and COLD1^Jap^ to RGA1 for regulation of [Ca^2+^]cyt level and GTPase activity^21^.

The *bZIP73*^*Jap*^ gene can be up-regulated by ABA treatment (Supplementary Fig. 4); however, bZIP73^Jap^ represses the two ABA biosynthesis genes *OsNCED3* and *OsNCED5* during cold treatment (Supplementary Fig. 8), leading to lower ABA levels (Fig. 3a). This indicates that *bZIP73*^*Jap*^ not only responds to ABA signaling, but is also a negative regulator of ABA synthesis and ABA signaling. At the same time, bZIP73^Jap^ *trans*-activates POX precursors under both normal and cold temperature conditions (Supplementary Fig. 8), thus acting as a positive regulator of ABA-dependent cold stress signaling pathways. Taken together, we propose that bZIP73^Jap^ is a bi-functional transcription factor with both repressing and activating activities to fine-tune ABA homeostasis and signaling to balance cold stress responses with requirements for growth and development. Similarly, it was shown that OsbZIP23 *trans*-activates the ABA biosynthesis gene *OsNCED4*, leading to higher ABA levels and amplified ABA signaling. Moreover, OsbZIP23 *trans*-activates the negative regulator of ABA signaling gene PP2C (*OsPP2C49*) and is also involved in fine-tuning of ABA signaling to promote drought tolerance^44^.

Cold tolerance is a very complex trait^3^. Over the past 20 years, extensive efforts were made to identify cold tolerance quantitative trait loci (QTLs) in rice, but only a few associated cold tolerance genes were cloned and functionally verified, such as *COLD1*^21^, *qCTS12/GSTZ2*^51^, *CTB4a*^22^, and *Ctb1*^52^. Here we show that the *japonica* allele of *bZIP73* associates with the LTSS cold tolerance phenotype of the rice mini-core population, and *bZIP71* is associated with the QTL of “survival rate after nature chilling stress” *Locus* 182 reported by Lv et al.^7^ and *qLTSS9-1* reported by Schläppi et al.^36^. This suggests that *bZIP71* and *bZIP73* are two cold tolerance QTL-associated genes that could be used for gene pyramiding to improve cold stress tolerance of rice cultivars.

Our evolutionary and population genetic analyses suggest that two specific alleles of *bZIP73* were repeatedly selected in both the *japonica* and *indica* subspecies of rice, indicating that balancing selection may have occurred for this gene (Fig. 7).

## Methods

### Association analysis and genome-wide selection screening

A total of 91 *bZIP* genes were collected from Nijhawan et al.^25^ and the Michigan State University (MSU) Rice Genome Annotation Project (RGAP)^26^. Whole-genome SNP data including 2.3 million markers were obtained from Wang et al.^34^. SNPs located within the coding sequence (CDS) of *bZIP* genes with missing data rates less than 80% were retained from the whole-genome dataset. Nucleotide variations of *bZIP* genes were translated to aa variations using a custom Perl script, which were further screened for minor allele frequency >1% and linkage less than 0.2 using PLINK^53^ with parameters *–indep-pairwise 5 2 0*.*2 –maf 0*.*01*. Stratification of the USDA mini-core population was performed using the thinned bZIP amino-acid data by PLINK^53^ with parameters *–cluster –ppc 1e-3 –mds-plot 3*. Gene association analyses with FDR being controlled in multiple testing (FDR_BH) were performed by PLINK^53^ using parameters of *–covar-number 1,2 –no-fid –no-parents –no-sex –linear –adjust*. LTSS phenotype data for mini-core accessions were retrieved from Schläppi et al.^36^.

A whole-genome scan for selective sweeps was carried out using OmegaPlus^38^ with parameters *-minwin 500 -maxwin 25000 -impute N -binary*, and the *-grid* parameter was determined by sequence length with 5 kb per grid. Centromeric regions^26^ with 500 kb extended on both sides were masked to control for abnormal LD. Regions with top 5% *ω* scores in a chromosome were considered being positively selected in the population. *bZIP* genes that overlapped with or fell into selective sweeps were considered positively selected.

### Sequences of *bZIP73* from wild species and other cultivars

*bZIP73* orthologs were identified from the genomes of *O*. *barthii*, *O. glumaepatula*, *O. glaberrima*, *O. longistaminata*, *O. meridionalis*, *O. punctata*, and *O. rufipogon* using BLAST search in Gramene (http://www.gramene.org). Another four orthologs of *O*. *nivara* were BLAST-searched from accessions IRGC 106105, IRGC 106154, IRGC 80470, and IRGC 89215 generated by Xu et al.^53^. The other *bZIP73* variances were representatives of different accessions of the USDA mini-core collection^34^ with IDs indicated in Fig. 5a in parentheses. These varieties were selected based on: (1) the major allele frequency (>80%) of the accession and (2) missing data rate (~6% in average). Multiple sequence alignment of the *bZIP73* sequences was performed using MEGA 6.0 with default parameters^54^.

### DNA constructs and rice transformation

Structural information for *bZIP73* was obtained from the Plant Transcription Factor Database (PlnTFDB)^55^. For the construction of *bZIP73* OE vectors, CDS of *bZIP73* genes were reverse transcribed from total RNA extracted from 2-week-old rice seedlings (*bZIP73*^*Jap*^ from Nipponbare; *bZIP73*^*Ind*^ from Kasalath) using PCR (Supplementary Data 5). The sequences were confirmed by PCR fragment sequencing and cloned into binary expression vector pCambia1300-221 to produce 3× Flag-fusion proteins expressed from the strong CaMV 35S promoter, resulting in vectors bZIP73^Jap^::Flag (73^Jap^OE) and bZIP73^Ind^::Flag (73^Ind^OE). For double overexpression of *bZIP71* and *bZIP73*^*Jap*^ (71–73^Jap^OE), the coding sequences of *bZIP71* and *bZIP73*^*Jap*^ were inserted into the binary expression vector pBWA(V)HS and pBWD(LA)1C, respectively, both of which carried the CaMV 35S promoter and nopaline synthase terminator. The pBWD(LA)1C-*bZIP73*^*Jap*^ construct functioned as the supply vector, and the pBWA(V)HS-*bZIP71* construct functioned as the donor vector. Using homologous recombination, the supply vector (pBWD(LA)1C-*bZIP73*^*Jap*^) was ligated into the donor vector (pBWA(V)HS-*bZIP71*), generating the pBWA(V)HS-*bZIP71*-*bZIP73*^*Jap*^ vector. To generate the *bZIP73*-RNAi vector, DNA fragments of *bZIP73* (–296 bp to +179 bp) consisting of a sense and antisense strand separated by an intron (the first intron of GA20 oxidase from potato) was inserted into the multiple cloning site of p2300Act (a derivative of pCAMBIA2300 carrying the *Actin* I promoter and the octopine synthase terminator)^56^. The OE and RNAi constructs were introduced into *Agrobacterium tumefaciens* strain AGL1 and transformed into the *japonica* rice cultivar (cv.) Zhonghua 11 (ZH11). All primer sequences are listed in Supplementary Data 5.

### Construction of *bZIP73*^*Ind*^ near-isogenic lines

A set of chromosome segment substitution lines were constructed by recurrent backcrossing between Teqing (*O. sativa* L. ssp. *indica*) as the donor parent and Lemont (*O. sativa* L. ssp. *japonica*) as the recurrent parent. From the BC_1_F_2_ to the BC_4_F_2_ generation, we continuously selected for target lines containing the recurrent parent markers by using both Simple-Sequence Repeats and Sequence-Tagged Sites markers, and finally a NIL containing *bZIP73*^*Ind*^ was identified in the BC_4_F_2_ generation using markers T176 and T183 (Supplementary Data 5). The NIL carried an ~615 kb Teqing genome segment containing *bZIP73*^*Ind*^, and the BC_4_F_6_ generation was used in this study.

### Plant growth conditions and stress treatments

All rice transgenic lines used in this study were derived from the *japonica* cv. ZH11. Seeds were surface sterilized with 70% ethanol for 3 min and soaked in water at 28 °C for 2 days in the dark, then grown hydroponically in the International Rice Research Institute (IRRI) (http://irri.org/) solution in a plant growth chamber (28 °C, 80% relative humidity, and a 14 h day/10 h night photoperiod). Two-week-old seedlings were treated with ABA (100 μM), H_2_O_2_ (2 mM), and cold stress (4 °C), respectively. Seedlings were sampled after 0, 0.5, 1, 3, 6, 12, and 24 h of treatment after which total RNA was extracted, and expressions of relative genes were determined using quantitative reverse transcription PCR (qRT-PCR).

For cold stress treatment at the seedling stage, homozygous transgenic lines of *bZIP73*^*Jap*^ OE, *bZIP73*^*Ind*^ OE, *bZIP71* OE, *bZIP71*-*bZIP73*^*Jap*^ OE (71–73^Jap^OE), *bZIP73*^*Jap*^ RNAi, *bZIP71* RNAi, wild-type ZH11, NIL (*bZIP73*^*Ind*^), and Lemont were used. Two-week-old seedlings were treated at 4 °C for 3 days or 8 °C for 6 days after which plants were allowed to recover at 28 °C for 7 days. All experiments were replicated at least three times. Statistical analyses were performed using the SAS program (http://www.sas.com).

### RNA extraction and quantification

Total RNA was prepared from rice tissues using the Trizol reagent (Invitrogen, USA) according to the manufacturer’s protocol. For RT-PCR, 1 μg of total RNA was used for omplementary DNA (cDNA) synthesis using the ReverTra Ace qPCR RT Master Mix and gDNA removal kit (Toyobo, Japan). The obtained cDNA library was used as template for real-time PCR analysis, which was carried out using the SYBR Premix Ex Taq Kit (Takara, Japan) protocol and the ABI PRISM 7900HT Sequence Detection System (Applied Biosystems, USA). The rice *Ubiquitin* gene (*LOC_Os03g13170*) was used as internal control. Primer sequences for qRT-PCR are listed in Supplementary Data 5. The experiments were repeated at least three times.

### Subcellular localization and *trans*-activation activity

Full-length CDS of *bZIP73*^*Jap*^ and *bZIP73*^*Ind*^ were inserted into pCambia2300-35S-GFP, creating bZIP73^Jap^::GFP and bZIP73^Ind^::GFP fusion vectors. Plasmid DNA was prepared using the Plasmid Midi Kits (Qiagen, Germany) according to the manufacturer’s instructions. Co-transformed bZIP73^Jap/Ind^::GFP and OsMADS3::mCherry vectors were expressed transiently in rice leaf protoplasts using the polyethylene glycol method^57,58^. After 12 h of incubation in the dark at 28 °C, green fluorescent protein (GFP) fluorescence was recorded using a confocal laser-scanning microscope (Carl Zeiss, LSM510, Germany). OsMADS3 was used as a nuclear localization marker^59^.

*Trans*-activation activity assays were performed using the Matchmaker GAL4 Two-Hybrid System 3 (Clontech, USA). Full-length coding sequences of *bZIP73*^*Jap*^, *bZIP73*^*Ind*^, and a series of truncated sequences of *bZIP73*^*Jap*^ and *bZIP73*^*Ind*^ were amplified, cloned, and fused to the GAL4 DNA-binding domain (BD) in vector pGBKT7 to generate GAL4 BD, BD-73^Jap/Ind^ (aa: 1–173), BD-73N1 (aa: 1–99), BD-73^Jap/Ind^C1 (aa: 100–173), BD-73N2 (aa: 1–156), BD-73M (aa: 77–156), and BD-73^Jap/Ind^C2 (aa: 77–173). All constructs and the empty vector were transformed into yeast strain AH109, serially diluted, and spread onto either tryptophan-deficient synthetic dropout medium, or tryptophan-, histidine-, and adenine-deficient synthetic dropout medium. Primer sequences for the constructs are listed in Supplementary Data 5.

### Measurement of *trans*-activation activity in vivo

Promoters of *OsNCED3*, *OsNCED5*, *LOC*_*Os01g22249*, *LOC*_*Os03g02920*, *LOC*_*Os03g32050*, and *LOC*_*Os04g59210* (~2000 bp upstream of these genes) were amplified and cloned into the pGreenII 0800-LUC vector containing the firefly luciferase (fLUC) gene and the *Renilla* LUC gene (rLUC) as reporters^60^, while *bZIP71* and *bZIP73* cDNAs were cloned into the pCambia1300-221-Flag vector as effectors. Assays for transient expression in rice protoplasts were then divided into two populations and incubated for 12 h in the dark. After incubation, one of the transformed protoplast populations was cold treated at 4 °C in the dark for 3 h. Finally, protoplasts were collected by centrifugation at 450 × *g* for 3 min and immediately utilized for luciferase assays. Luciferase activity was quantified using a dual-luciferase reporter assay kit (Promega, E1910, USA). Five independent transformations for each sample were performed, and the relative luciferase activity was calculated as the ratio of fLUC to rLUC (fLUC/rLUC).

### Yeast two-hybrid assays

cDNA library from various tissues (including roots and shoots at the seedling stage; roots, leaves, stems, flowers, and seeds at the reproductive stage) of rice plants was constructed and detection of interacting proteins was performed using the Make Your Own Mate & Plate™ Library System (Clontech, 630490, USA) and Matchmaker Gold Yeast Two-Hybrid System (Clontech, 630489, USA) following the manufacturer’s instruction. The final prey library was transformed into the yeast strain Y187 following the Yeast Protocols Handbook (Clontech, PT3024-1, USA). The CDS of *bZIP73*^*Jap*^ was cloned into the pGBKT7 vector and functioned as bait. Bait constructs were introduced into yeast strain Y2H Gold (Clontech, 630489, USA). Mating-based yeast two-hybrid assays were performed according to the manufacturer’s instruction (Clontech, 630489, USA) using haploid yeast strains, Y2H Gold and Y187, for bait and prey, respectively. For identification of interaction proteins of bZIP71 and bZIP73^Jap/Ind^ in yeast, the CDS of *bZIP71* was cloned into the pGBKT7 vector and functioned as bait; the CDS of *bZIP73*^*Jap*^ and *bZIP73*^*Ind*^ were, respectively, cloned into the pGADT7 vector and functioned as prey. Bait and prey vectors were co-transformed into the yeast strain Y2H Gold, then incubated on SD/Leu-Trp- plates at 30 °C for 2 days. Positive clones were cultivated on fresh SD/His-Ade-Leu-Trp- plates at 30 °C for 2 to 4 days. β-Galactosidase activity was measured using a liquid culture assay with *o*-Nitrophenyl-β-d-Galactopyranoside (ONPG, Amresco, USA) as substrate according to the Yeast Protocols Handbook (Clontech, PT3024-1, USA). Primer sequences for vectors are listed in Supplementary Data 5.

### Bimolecular fluorescence complementation

To perform BiFC assays, CDS of *bZIP73*^*Jap*^ and *bZIP73*^*Ind*^ were cloned into the pSPYNE vector to construct bZIP73^Jap^::NYFP and bZIP73^Ind^::NYFP, and the *bZIP71* CDS was cloned into the pSPYCE vector to generate bZIP71::CYFP^61^. Plasmids were transformed into rice protoplasts by polyethylene glycol-mediated transformation^58,61^. GFP fluorescence was visualized with a confocal scanning microscope (Carl Zeiss, LSM510, Germany) after incubation in the dark for 14 h. Primer sequences for vectors are listed in Supplementary Data 5.

### Co-immunoprecipitation

To generate epitope-tagged expression vectors for Co-IP, bZIP73^Jap^::Flag and bZIP73^Ind^::Flag constructs were modified by replacing the 3× Flag with a 6× Myc tag, resulting in bZIP73^Jap^::Myc and bZIP73^Ind^::Myc vectors, respectively. These constructs were introduced into *Agrobacterium tumefaciens* (AGL1) and transformed into a single-copy homozygous overexpression line of bZIP71::Flag^62^. The Co-IP constructs were also transformed into single-copy homozygous transgenic lines of pCambia1300::Flag (empty binary vector) as the negative control. Soluble proteins were extracted from transgenic lines and Co-IP assays were performed following the protocol of the FLAG Immunoprecipitation Kit (Sigma, FLAGIPT1, USA). Precipitations were visualized on sodium dodecyl sulfate–polyacrylamide gel electrophoresis (SDS-PAGE) using Anti-Flag (Sigma, F1804, USA), Anti-Myc (Sigma, M4439, USA), and horseradish peroxidase (HRP)-conjugated anti-mouse (Promega, W4021, USA) antibodies. All primary antibodies were diluted 1:1000. Primer sequences for vectors are listed in Supplementary Data 5. The original blot scans are shown in Supplementary Fig. 13.

### Pull-down assays

The CDS of *bZIP71* was cloned into plasmid pGEX4T-3 as a glutathione *S*-transferase (GST)-fusion protein (GST::bZIP71); *bZIP73*^*Ind*^ was subcloned into maltose-binding protein (MBP) tag containing plasmid pMALc2x to generate fusion protein MBP::bZIP73^Ind^. The two fusion proteins were expressed in *Escherichia coli* BL21. *bZIP73*^*Jap/Ind*^ was subcloned into polyhistidine (His) containing plasmid pET32a to generate fusion proteins of His::bZIP73^Jap/Ind^, and fusion proteins were expressed and purified in vitro according to the protocol of the TnT Quick Coupled Transcription/Translation Systems (Promega, USA). Pull-down assays were performed using the MagneGST Pull-Down System (Promega, USA) following the manufacturer’s protocol. For pull-down assays, 2 μg of His::bZIP73^Jap^ or His::bZIP73^Ind^ prey proteins were incubated with immobilized GST (2 μg) or GST::bZIP71 (2 μg) bait proteins. For competitive pull-down assays, 2 μg His::bZIP73^Jap^ and 2, 5, 10 μg MBP::bZIP73^Ind^ prey proteins were incubated with immobilized GST (2 μg) or GST::bZIP71 (2 μg) bait proteins. Precipitates were washed three times and visualized on SDS-PAGE using Anti-His (GE Healthcare, 27-4710-01, UK), Anti-GST (Beijing Protein Innovation, AbM59001-2H5-PU, China), Anti-MBP (New England Biolab, E8032, USA), and HRP-conjugated anti-mouse (Promega, W4021, USA) antibodies. All primary antibodies were diluted 1:1000. As a control, 10% of precipitation inputs were visualized directly on SDS-PAGE. Primer sequences are provided in Supplementary Data 5. The original blot scans are shown in Supplementary Fig. 13 and 14.

### Chromatin immunoprecipitation assays

To perform ChIP assays, 2-week-old seedlings of *bZIP73*^*Jap*^::Flag OE, *bZIP73*^*Ind*^::Flag OE, and *bZIP71*::Flag OE lines were harvested and immediately fixed with 1% formaldehyde for chromatin isolation. DNA was immunoprecipitated using anti-Flag antibodies and purified using the PCR Purification Kit (Qiagen, Germany)^44^. For ChIP-sequencing, the precipitated and purified DNA from *bZIP73*^*Jap*^::Flag OE lines were used to generate sequencing libraries, which were sequenced and processed by Boao Biological Co., Ltd (Beijing, China), generating 19 million 50 bp single-end reads. Raw reads were cleaned and trimmed using a custom Perl script and mapped to the rice reference genome (MSU TIGR v7)^26^. The Model-based Analysis of ChIP-Seq (MACS) software^63^ was used to identify chromatin interaction peaks with default parameters. Annotation of the peaks was done using a custom Perl script and all transposon genes were removed. GO enrichment analysis was performed and visualized using the RiceNetDB database^64^.

For quantifications using real-time PCR (ChIP-qPCR), the amount of precipitated DNA was calculated relative to the total input chromatin and expressed as percentage of the total according to the formula: % input = 2^Δ*C*t^ × 100%, where Δ*C*t = *C*t (input)—*C*t (IP), where *C*t is the mean threshold cycle of the corresponding PCR reaction. The average expression level was obtained from the percent input values of three replicates^65^. Primer sequences for ChIP-qPCR are listed in Supplementary Data 5.

### Electrophoretic mobility shift assays

CDS of *bZIP71*, *bZIP73*^*Jap*^, and *bZIP73*^*Ind*^ were amplified and cloned into the pGEX4T-3 vector to generate GST fusion proteins. Vectors of *bZIP71*::GST and *bZIP73*^*Jap*^::GST were expressed in *E. coli*, which were purified following the protocol of the MagneGST Protein Purification System (Promega, USA). The bZIP73^Ind^::GST vector was expressed and purified using the TnT Quick Coupled Transcription/Translation Systems according to the manufacturer’s protocol (Promega, USA). For promoter binding experiments, complementary single-stranded oligonucleotides derived from 70 bp of the G-box region of gene promoters were synthesized as DNA probes. To obtain double-stranded G-Box motif-containing fragments, two complementary oligonucleotides were mixed in a water bath at 95 °C for 5 min and cooled to room temperature for annealing. Mutated G-box was amplified from wild type using mutated primers and was used as negative control. GST fusion protein binding experiments were performed in vitro with the Chemiluminescent EMSA Kit (Beyotime, China) using standard protocols. Primer sequences used are listed in Supplementary Data 5.

### Abscisic acid and reactive oxygen species quantifications

Peroxidases were extracted in 0.05 M potassium phosphate buffer (pH 7.0) and subsequently centrifuged at 4 °C, 12,000 × *g* for 15 min. The supernatant was used as the enzyme extract. Measurement of POX activity was performed according to the protocol of the Peroxidase Activity Assay Kit (Beyotime, China).

To quantify endogenous ABA, 200 mg of freeze-dried 2-week-old seedlings were extracted twice with 7.5 mL of plant hormone extraction buffer (methanol/water/glacial acetic acid, 80:19:1, v/v/v) supplemented with 20 ng mL^−1^_2_H^6^ABA internal standards^44,66^. Quantifications were performed using an ABI 4000 Q-Trap (Applied Biosystems, USA). ROS examination was carried out using 14-day-old leaves sampled from seedlings with or without cold treatments. The samples were infiltrated with nitroblue tetrazolium (NBT) (Amresco, USA; 1 mg mL^−1^ NBT in 10 mM sodium azide and 10 mM phosphate buffer, pH 7.8) or 3, 3’-diaminobenzidine (DAB) solutions (Sigma-Aldrich, USA; 1 mg mL^−1^ DAB-HCl, pH 3.8)^67^. Stained leaves were imaged after removal of chlorophylls by boiling in 95% ethanol for 10 min. H_2_O_2_ content in seedlings was detected following the instruction of the Hydrogen Peroxide Assay Kit (Beyotime, China).

### Phylogenetic reconstruction of *bZIP73*

A 6-kb SNP dataset spanning *bZIP73* (MSU TIGR v7 reference coordinate: Chr9:18121580..18127580) was obtained from the combined population. A total of 452 varieties and accessions of cultivated rice and common wild rice were retained in the dataset after screening for per base missing data rate ≤20% and sequence missing data rate ≤20%. Duplicated sequences from different samples were removed to reduce redundancy. The homologous sequence of the 6-kb region obtained from *O. meridionalis* was used as outgroup (ROOT). A neighbor-joining (NJ) tree was reconstructed by MEGA 6^54^ using the Jukes–Cantor substitution model with gamma distribution adjustment. A total of 10,000 bootstrap pseudo-replications were conducted; however, due to the lack of diversity in the population sequences, bootstrap supports for branches were very low, indicating that the relationship between branches were not resolved. Thus, bootstrap values were not shown on the NJ tree. Visualization of the NJ tree was done using FigTree v1.4.3 (http://tree.bio.ed.ac.uk/software/figtree/).

### Reconstruction of the *bZIP73* haplotype network

Sequences of the 6 kb dataset (missing data rate <30%) were used. Cultivars with admixture backgrounds shown by population structure^18^ were removed to reduce noise. A total of 252 sequences were retained with ambiguous loci being imputed using NETWORK 5 (http://www.fluxus-engineering.com). A total of 98 haplotypes were identified after the sequence imputation. The MSN of all haplotypes was constructed using Arlequin 3.5.1.2^68^, visualized using HapStar^69^, and annotated using Inkscape (www.inkscape.org).

Estimation of haplotype scores was based on the 6 kb SNP markers with minor allele frequency ≥0.2. Genotypes of A, T, C, and G were scored as 1, 2, 3, and 4, respectively, and then the haplotype score was represented by the average of all marker scores. Ortholog sequences of *bZIP73* from wild rice relatives were used to estimate the ancestral haplotype score distribution of *bZIP73*. The 6 kb regions of *O. rufipogon* accessions and cultivated rice varieties were used to estimate the progenitor haplotype score distribution and the modern haplotype score distribution, respectively.

*O. rufipogon* samples shown in Fig. 6 were screened for unambiguous +511 SNP genotypes. Global Positioning System (GPS) information of each sample was obtained from Huang et al.^14^ and Wang et al.^34^. Maps for sample distributions were created using ArcGIS^®^ software from the Environmental Systems Research Institute, Inc. (Esri). The map layer of terrestrial temperature averages from 1960 to 1990 A.D. was obtained from ArcAtlas™ (www.arcgis.com).

### Nucleotide diversity of *bZIP73*

A 20-kb sequence set (Chr9:18112580..18133465) centered on *bZIP73* was obtained from the whole-genome dataset with a missing data rate of each sample ≤80%. The whole population was divided into subpopulations representing each rice accession based on the whole-genome phylogenetic tree by Wang et al.^34^. Nucleotide diversity (Tajima’s *π*) of each accession was estimated using the script of ThetaPi_v2.pl (https://github.com/oushujun/ThetaPi)^70^ with parameters *-m0*.*8 -w100 -s0*. To detect the local reduction of nucleotide diversity, multiple comparisons of *π* values of the 4 kb region centered on *bZIP73*, the upstream region, and the downstream region were carried out in each subpopulation. The Ryan-Einot-Gabriel-Welsch Q (REGWQ) test and Fisher’s LSD test were applied for non-significant and significant analysis of variance results, respectively. The Satterthwaite approximation was applied for unequal pool sizes. The Kenward–Roger method was applied for unequal variances, and a log transformation was applied where unequal variances could be improved. Statistical analyses were performed using SAS.

### Data availability

ChIP-Seq data that support the findings of this study have been deposited to the NCBI Sequence Read Archive (SRA) under BioProject PRJNA450934. All other relevant data are freely available from the corresponding author upon request.

## Electronic supplementary material

Supplementary Information

Description of Additional Supplementary Files

Supplementary Data 1

Supplementary Data 2

Supplementary Data 3

Supplementary Data 4

Supplementary Data 5
