# Supplementary material for: Early selection of bZIP73 facilitated adaptation of japonica rice to cold climates
Source: Nat Commun. 2018 Aug 17;9:3302. doi: 10.1038/s41467-018-05753-w (PMC6098049; doi:10.1038/s41467-018-05753-w)
Supplement: Supplementary file 1 — Supplementary Information [file 41467_2018_5753_MOESM1_ESM.docx]

**Early selection of *bZIP73* facilitated adaptation of *japonica* rice to cold climate**

Liu and Ou *et al*.

**
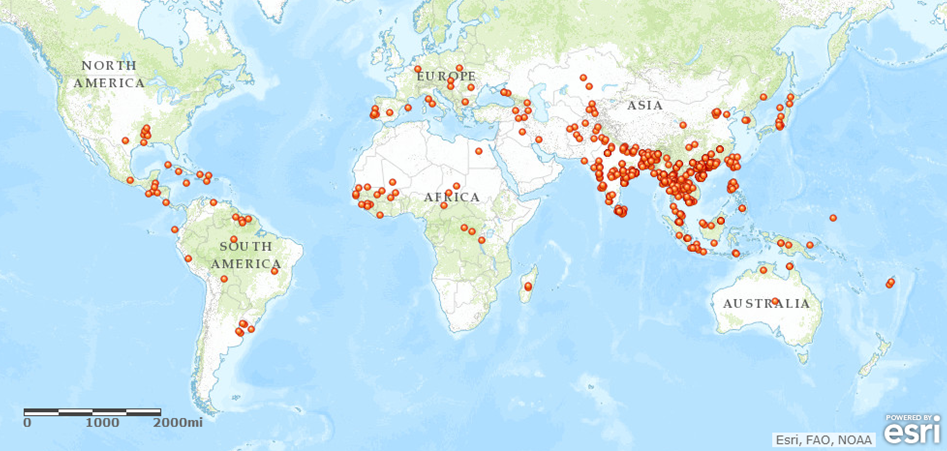
**

# **Supplementary Figure 1.** Geographic distribution of the USDA mini-core population and the wild rice population. Each red dot represents the location where a sample was collected. Global Positioning System (GPS) information was obtained from as reported previously by Wang *et al.*^1^ and Huang *et al.* ^2^. Copyright © Esri. All rights reserved.


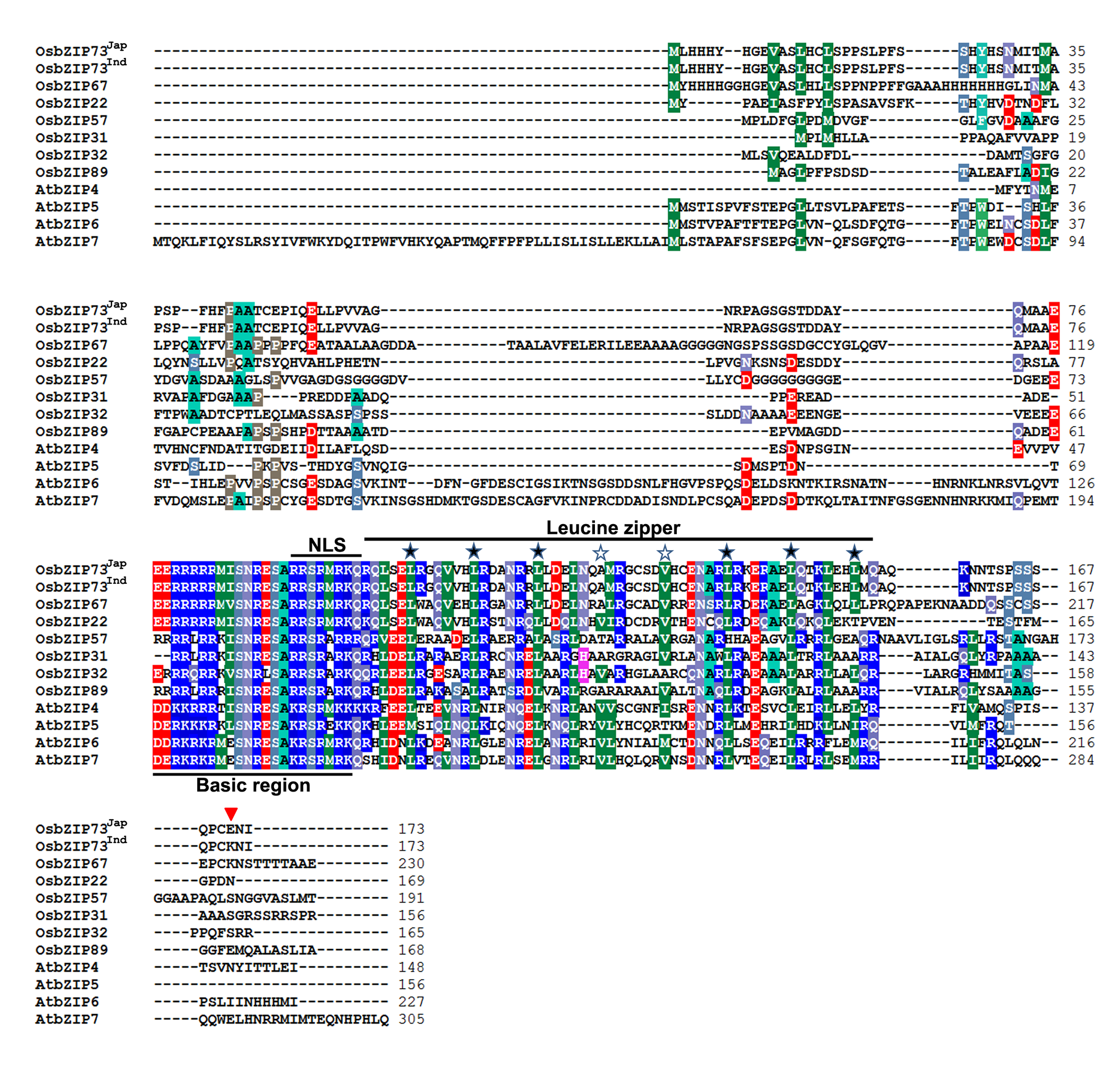


# **Supplementary Figure 2.** Sequence comparison of rice and Arabidopsis Group S3 bZIP proteins. NLS, nuclear localization signal. The two definitive domains, the basic region, and the leucine zipper, are indicated by lines. The conserved eight-leucine residues in the leucine zipper domain is indicated by stars. Red triangle indicates the amino-acid polymorphism between bZIP73^Jap^ and bZIP73^Ind^.


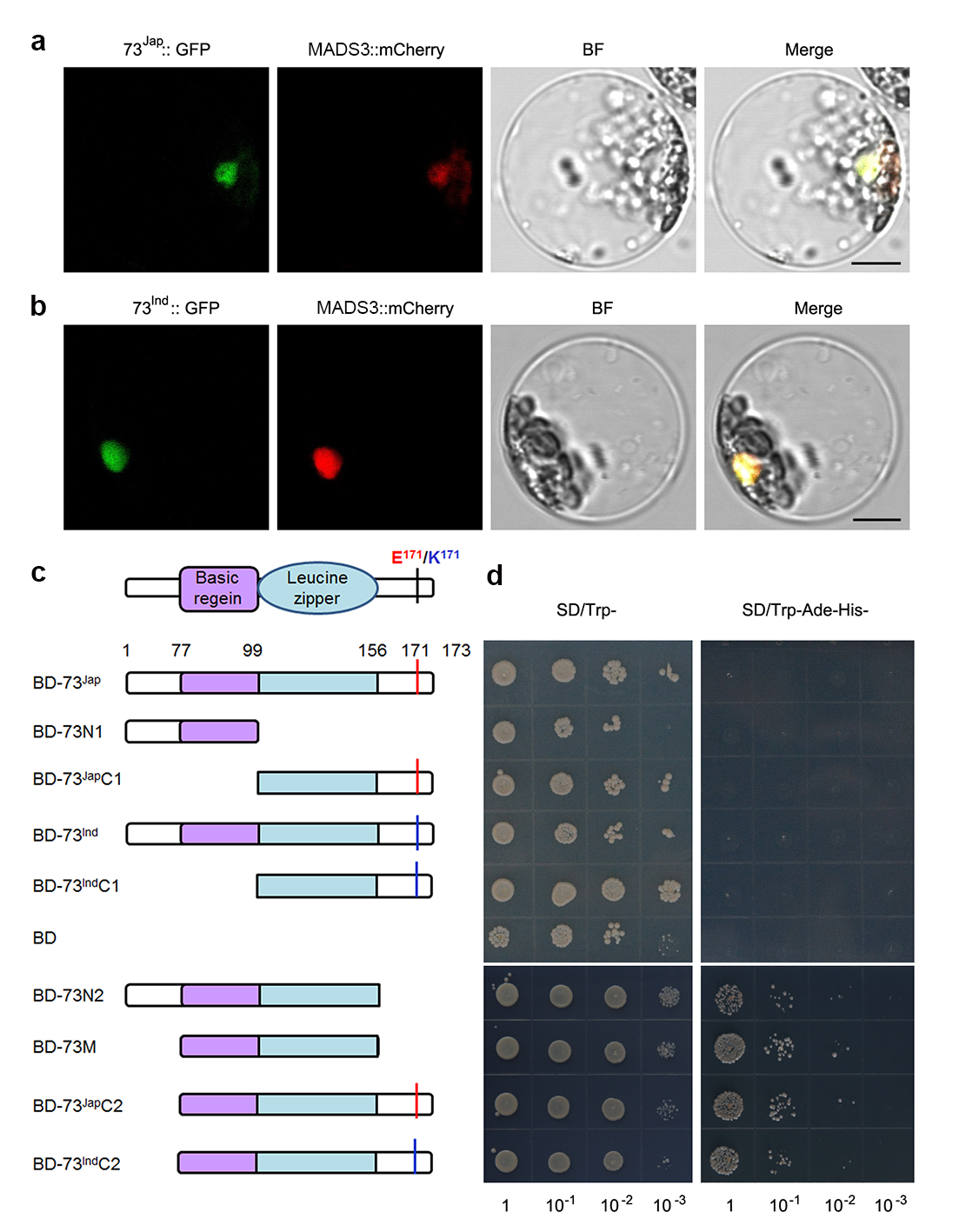


**Supplementary Figure 3.** Cellular localization and transactivation assays of bZIP73. (**a-b**) Cellular localization of the bZIP73^Jap^ (**a**) and bZIP73^Ind^ (**b**) protein in rice protoplasts. The fusion protein OsMADS3::mCherry was used as nucleus indicator. BF, bright field; Merge, overlay of the GFP, RFP and bright field images. Scale bars, 10 μm. (**c**) Constructs for transactivation assays of full-length and truncated bZIP73. Fusion proteins of the GAL4 DNA-binding domain (BD) and different portions of bZIP73 were cloned into the pGBKT7 vector, resulting in BD-73^Jap^ (1-173 aa), BD-73^Ind^ (1-173 aa), BD-73N1 (1-99 aa), BD-73^Jap^C1 (100-173aa), BD-73^Ind^C 1 (100-173aa), BD-73N2 (1-156 aa), BD-73M (77-156 aa), BD-73^Jap^C1 (77-173aa) and BD-73^Ind^C 2 (77-173aa) constructs. The pGBKT7 (BD) vector containing the GAL4 BD was used as a negative control. The two typical protein domains of bZIP73, basic region and leucine zipper, were indicated in purple and blue colors, respectively. The amino acid (aa) coordinate of bZIP73 is shown on top of the constructs. The amino acid variation at position 171 is shown using red (bZIP73^Jap^) and blue (bZIP73^Ind^) bars. (**d**) Transactivation assays of full-length and truncated bZIP73. Fusion transformants of the yeast strain AH109 were grown on SD/Trp- and SD/Trp-Ade-His- plates.

**
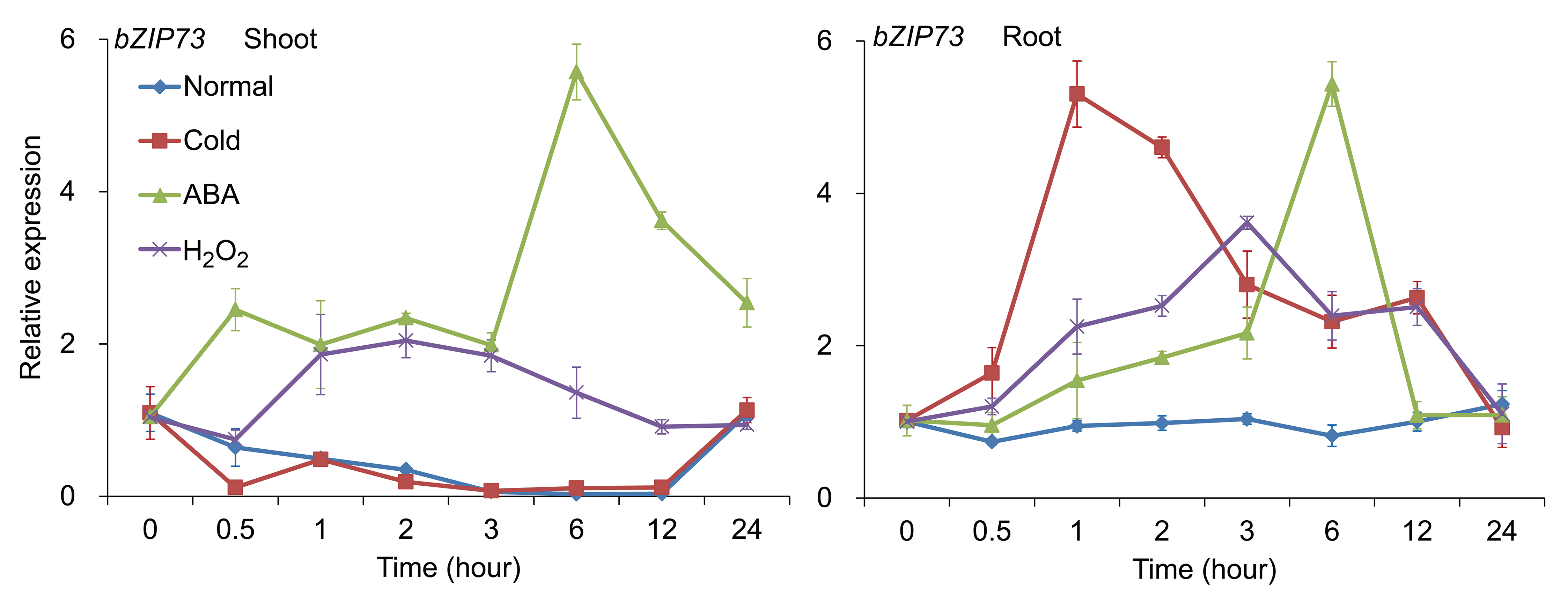
**

# **Supplementary Figure 4.** Relative expressions of *bZIP73^Jap^* under treatments of cold (4℃), ABA, and H_2_O_2_ in shoots and roots of wild-type Zhonghua 11 rice seedlings. Error bar, standard deviation from three independent experiments. Plants grown in the normal condition were used as untreated control.


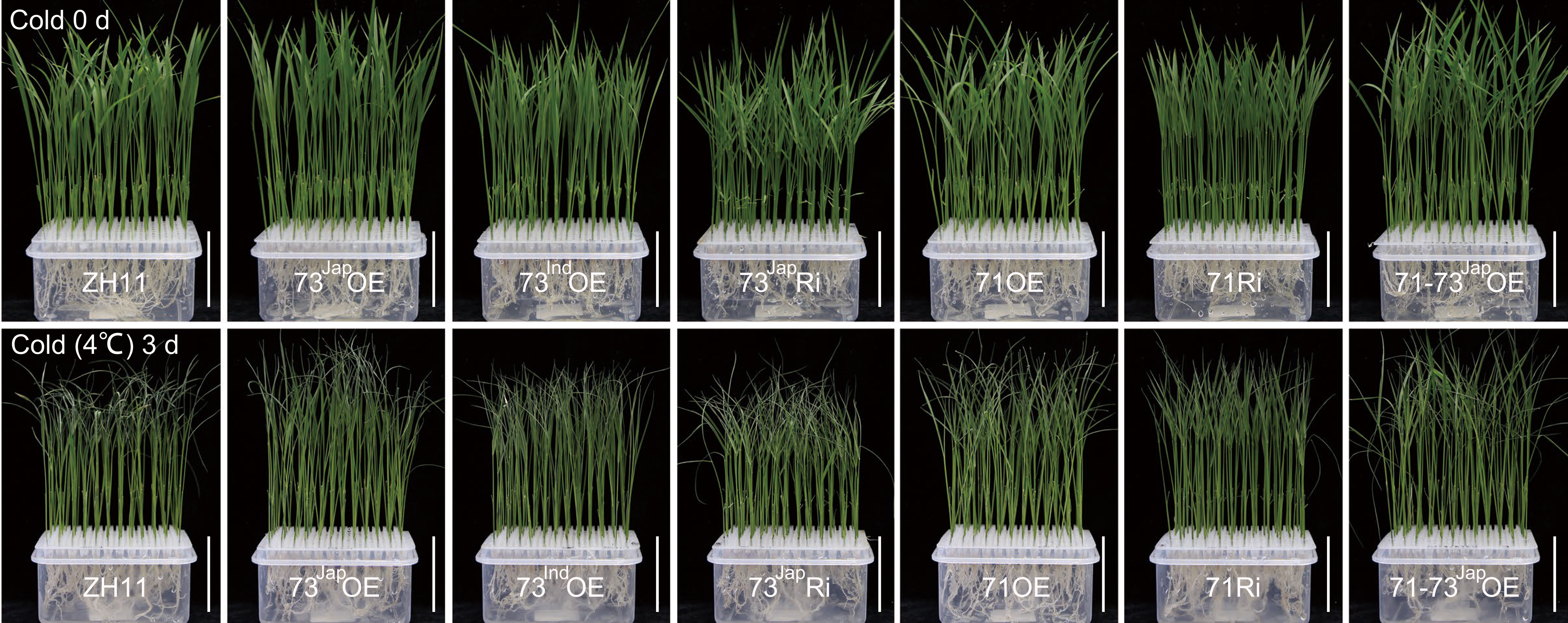


# **Supplementary Figure 5.** Assessing cold stress (4 ℃) tolerance of *bZIP73^Jap^*^/^*^Ind^* and *bZIP71* transgenic rice lines. Upper panel, seedlings before cold treatment; lower panel, seedlings immediately after treated in 4 ℃ for three days. ZH11, wild-type Zhonghua 11; 73^Jap^OE, *bZIP73^Jap^* overexpression lines; 73^Ind^OE, *bZIP73^Ind^* overexpression lines; 73^Jap^Ri, *bZIP73^Jap^* RNAi lines; 71OE, *bZIP71* overexpression lines; 71Ri, *bZIP71* RNAi lines; 71-73^Jap^OE, *bZIP73^Jap^* and *bZIP71* co-overexpression lines. Scale bars, 5 cm.

**
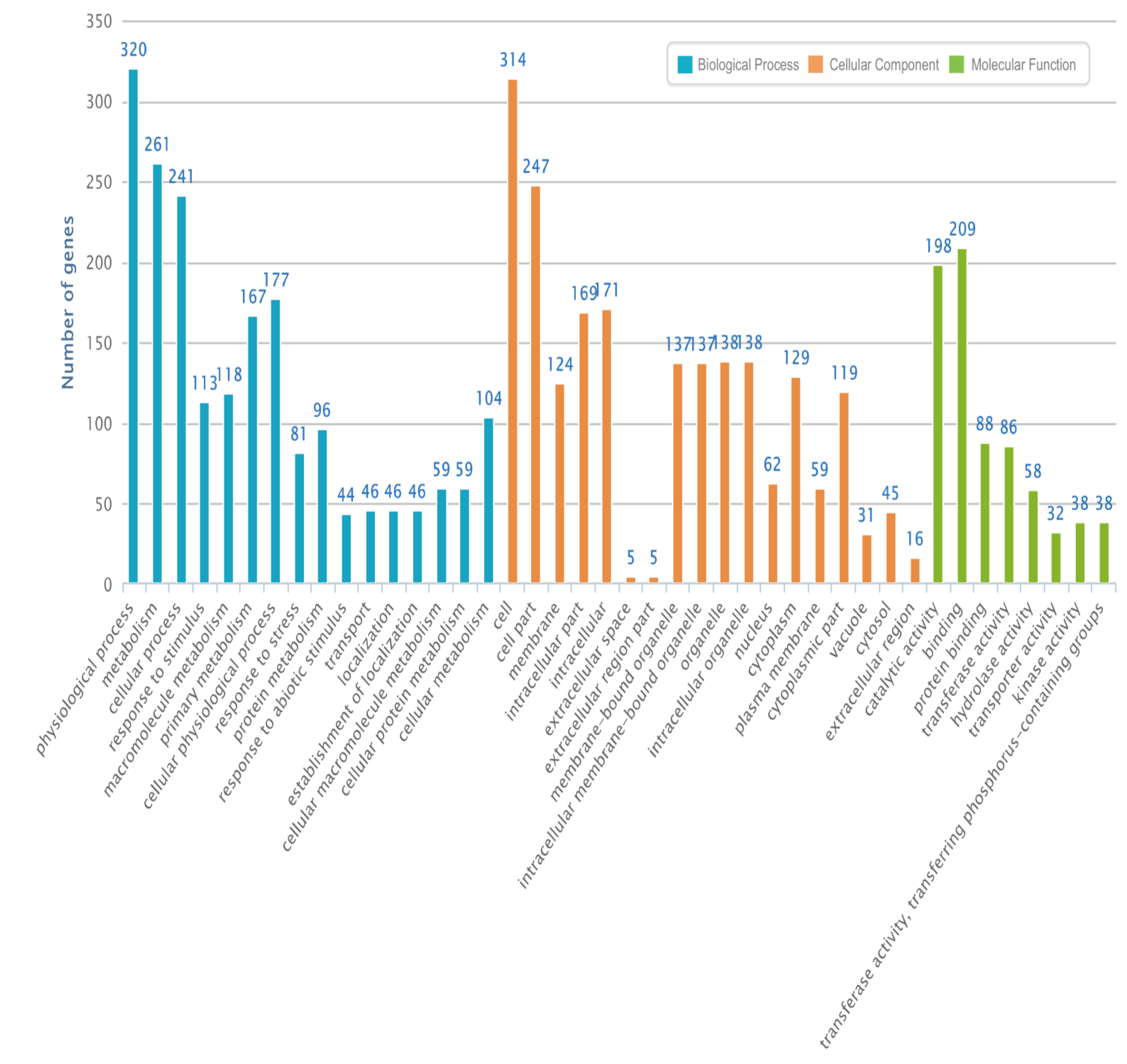
**

# **Supplementary Figure 6.** Gene ontology (GO) enrichment of genes precipitated with bZIP73^Jap^::Flag identified by ChIP-Seq. Number on each bar represent gene numbers in the respective GO term. The enrichment analysis and visualization were performed using the RiceNetDB database^3^.


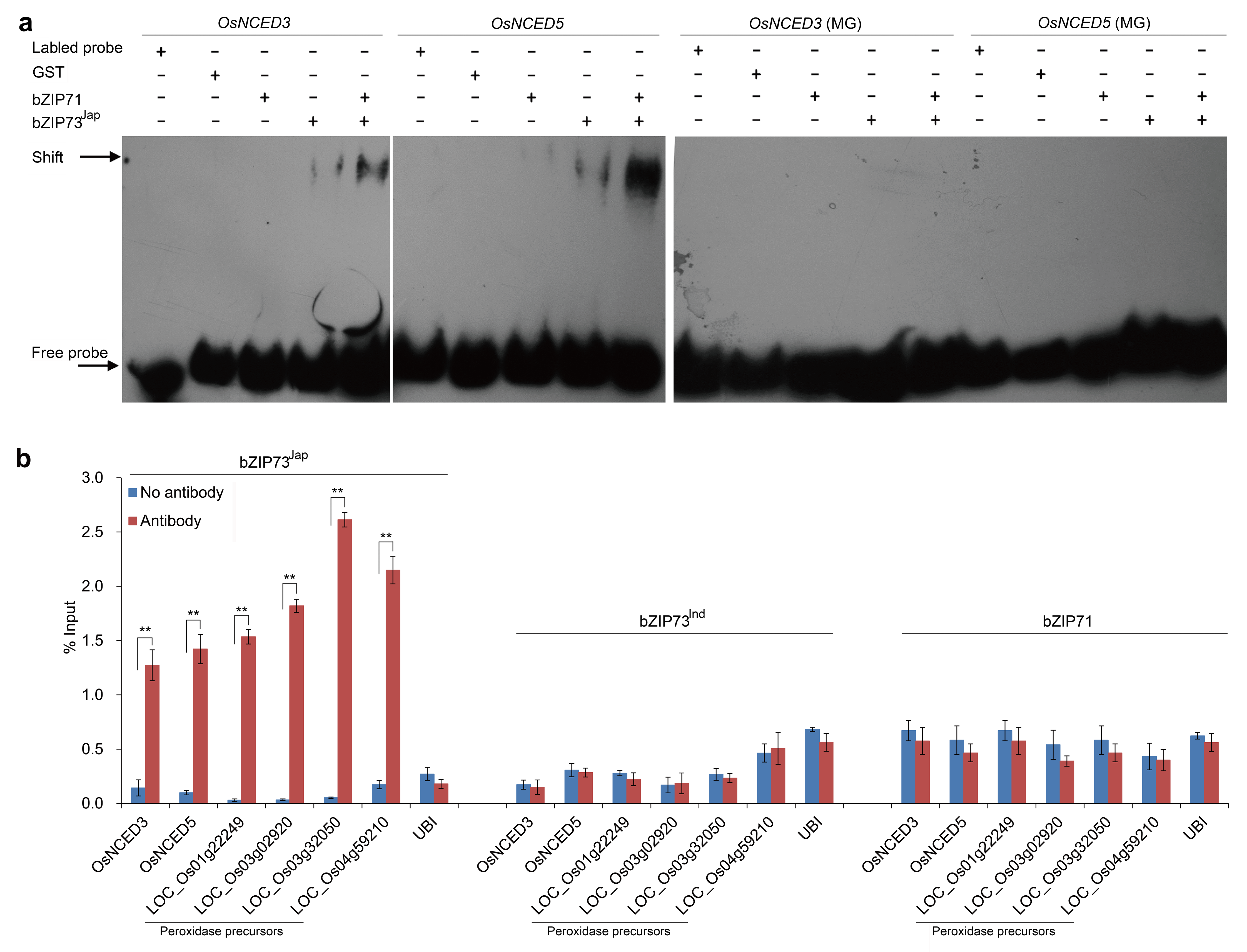


# **Supplementary Figure 7.** Binding interaction of bZIP73^Jap^ to the promoters of ABA biosynthetic genes and POX precursors. (**a**) *In vitro* EMSA using G-box sequences from promoters of *OsNCED3* and *OsNCED5* as probes. Left two panels, wild-type G-box sequences. Right panel, mutated G-box (MG) sequences. EMSA, electrophoretic mobility shift assay; Labeled probe, positive control; GST, negative control. (**b**) ChIP-qPCR using bZIP73^Jap^::Flag, bZIP73^Ind^::Flag, and bZIP71::Flag overexpression lines with three biological replicates. The anti-Flag antibody was used to precipitate bZIP73^Jap/Ind^/bZIP71-DNA interactions. Precipitated DNA was amplified with primers overlapping the G-box motif. ChIP, chromatin immunoprecipitation. Error bar, standard deviation. ***p* < 0.01, two-tailed *t*-test.


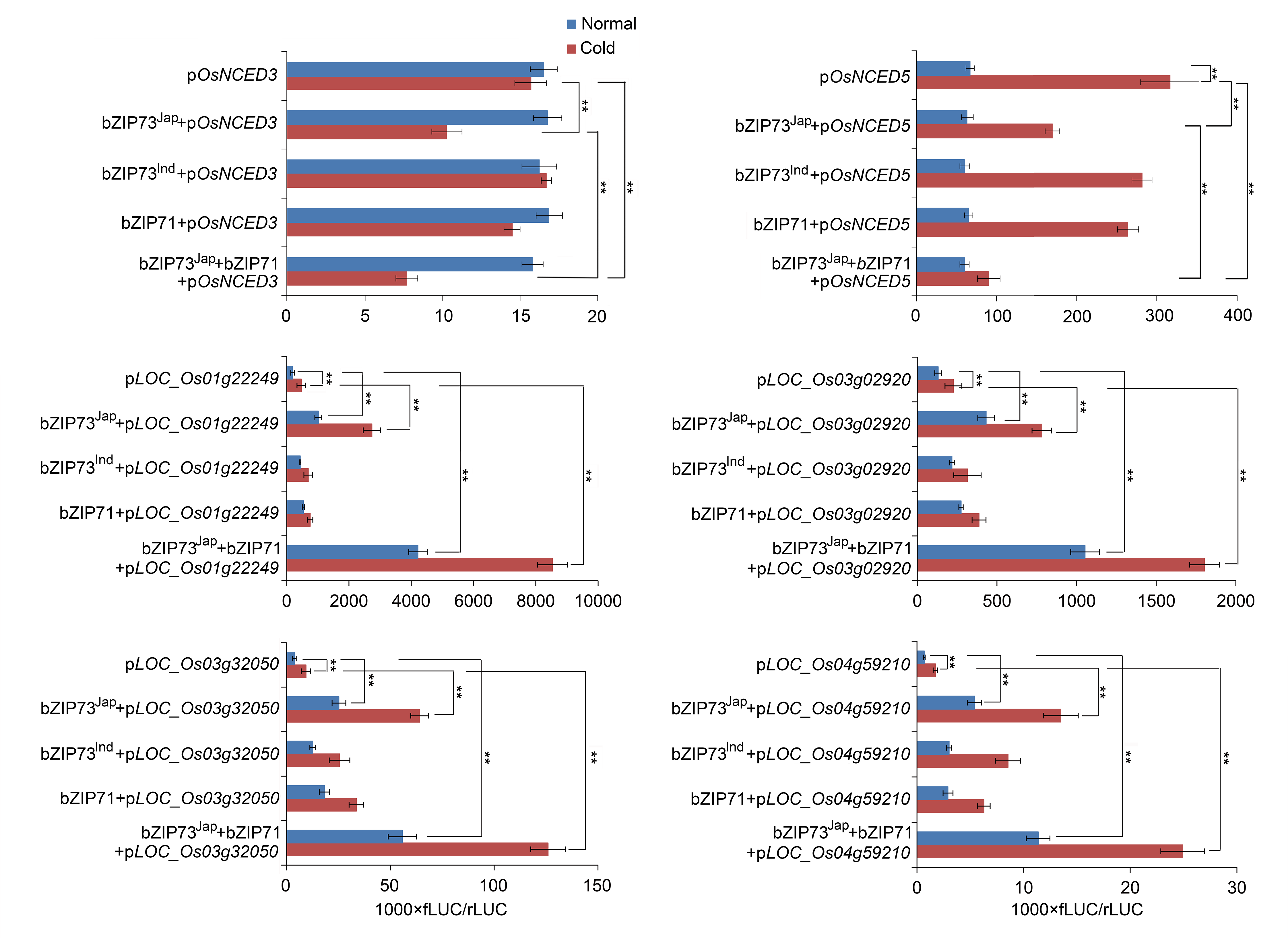


# **Supplementary Figure 8.** Relative promoter activity of ABA biosynthetic genes and peroxidase (POX) precursor genes under normal and cold conditions in rice protoplasts. The relative fLUC/rLUC ratio indicated by x-axes represents the relative promoter activity. Constructs used for each transfections in rice protoplasts as indicated by y-axes. Promoters of NCED genes and POX precursor genes were fused with LUC genes as reporters. After transfection, half of the protoplasts were incubated under cold condition (4 ℃, 3 h). Values are the mean of five independent replicates and error bars indicate SD. ***p* < 0.01, two-tailed *t*-test.


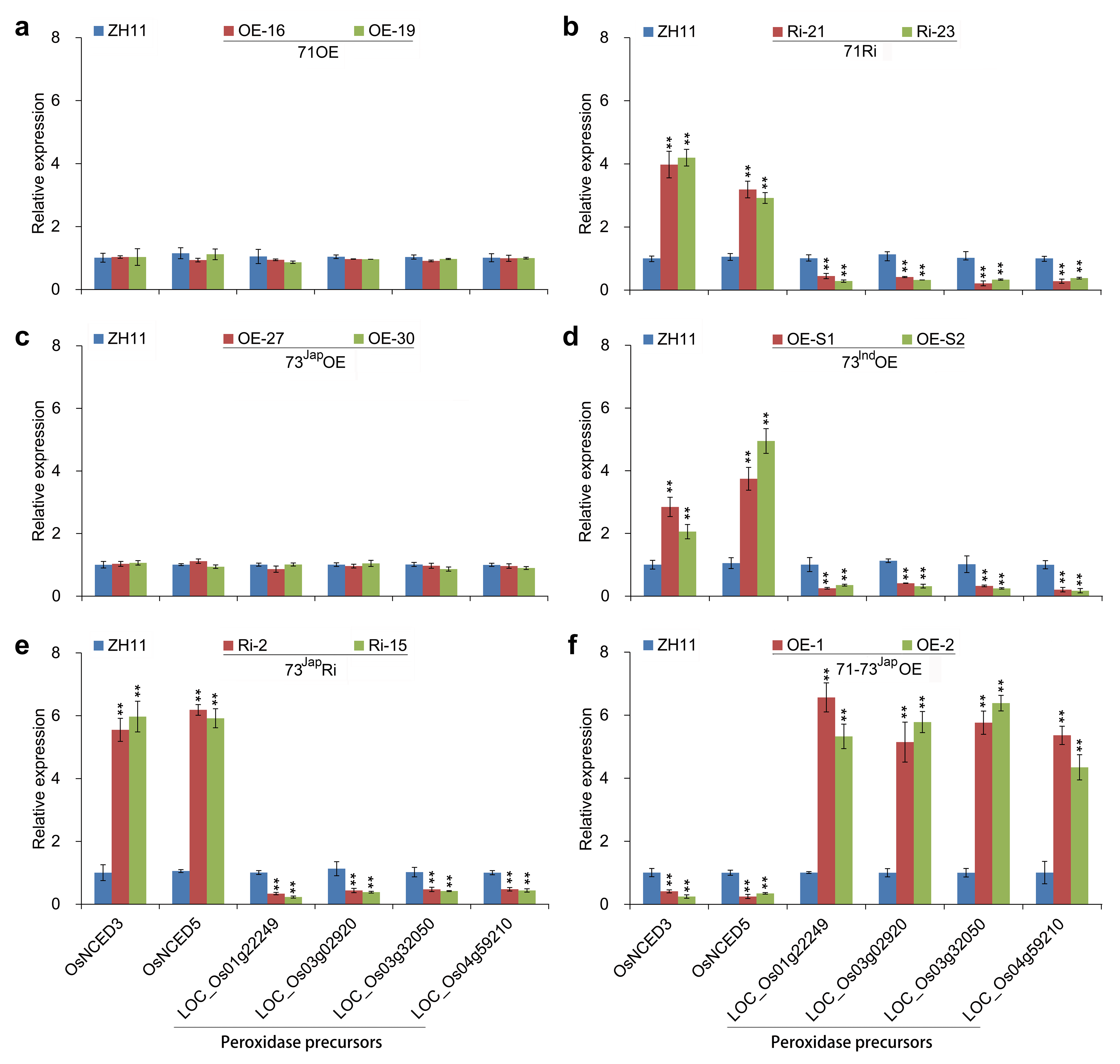


# **Supplementary Figure 9.** Relative expression levels of ABA biosynthetic and peroxidase precursor genes in (**a**) *bZIP71* overexpression (71OE) lines, (**b**) *bZIP71* RNAi (71Ri) lines, (**c**) *bZIP73^Jap^* overexpression (73^Jap^OE) lines, (**d**) *bZIP73^Ind^* overexpression (73^Ind^OE) lines, (**e**) *bZIP73^Jap^* RNAi (73^Jap^Ri) lines, and (**f**) *bZIP73^Jap^* and *bZIP71* co-overexpression (71-73^Jap^OE) lines as detected by qPCR. ***p* < 0.01, two-tailed *t*-test. Error bar, standard deviation of three biological replications (10 plants/replication).


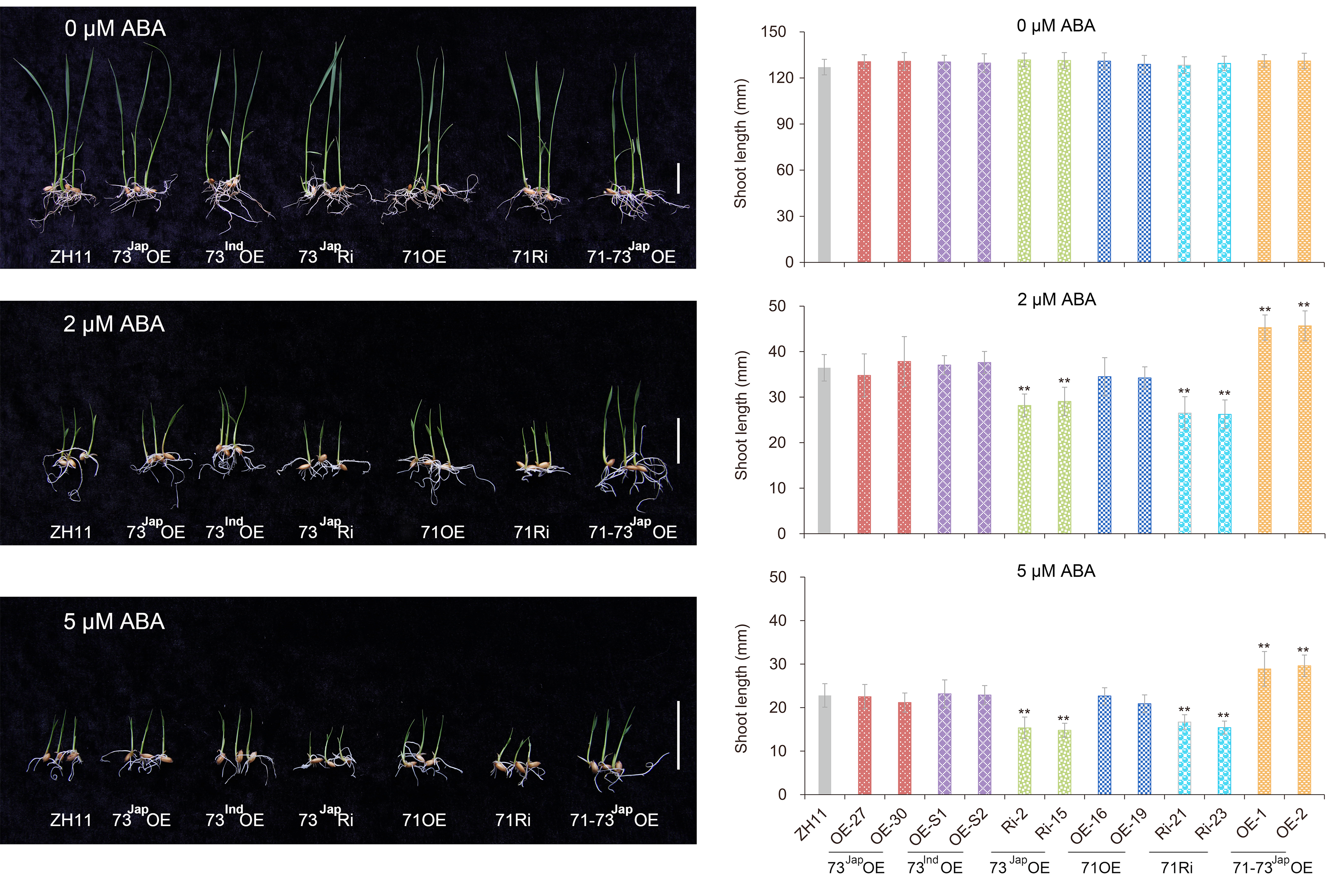


# **Supplementary Figure 10.** *bZIP73^Jap^* OE (73^Jap^OE) and *bZIP71* RNAi (71Ri) lines were hypersensitive to ABA treatment; co-expression lines of *bZIP71*-*bZIP73^Jap^* (71-73^Jap^OE) lines were less sensitive to ABA treatment. Wild-type Zhonghua 11 (ZH11), *bZIP73^Ind^* (73^Ind^OE), *bZIP71* (71OE), 73^Jap^OE, 71-73^Jap^OE, *bZIP73^Jap^* RNAi (73^Jap^Ri), and 71Ri lines were treated with ABA (2 and 5 µM) for 10 days. Right panel, average shoot lengths of seedlings with three biological replications (30 seedlings/replication). Error bar, standard deviation. Scale bars, 3 cm. ***p* < 0.01, two-tailed *t*-test.


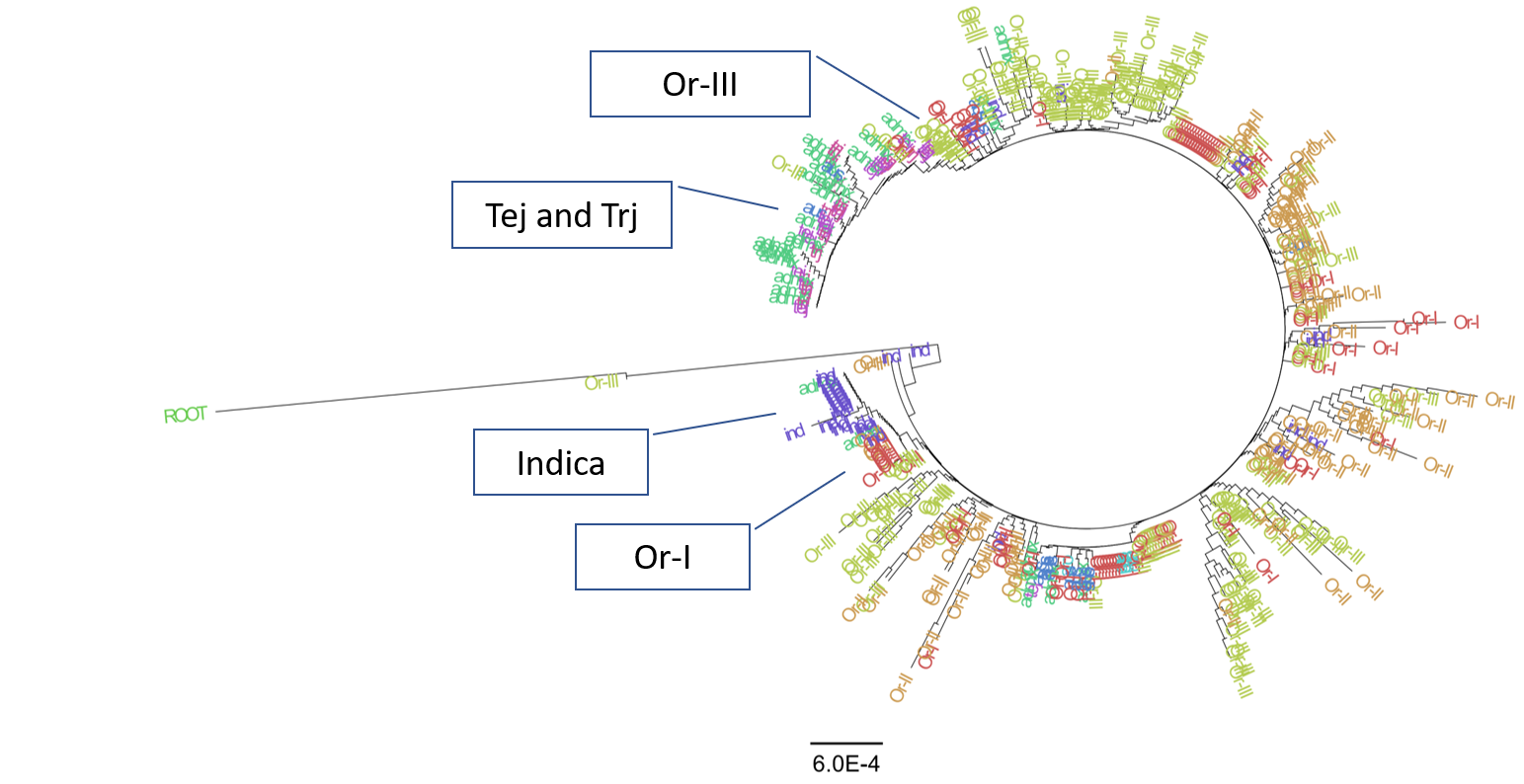


# **Supplementary Figure 11.** The two alleles of *bZIP73* were derived from different common wild rice progenitors. The phylogeny was reconstructed using 6 kb sequences flanking *bZIP73* with the neighbor-joining method. Tej, *temperate* *japonica*; Trj, *tropical japonica*; Ind, *indica*; Aus, *aus*; Aro, *aromatic*; Admix, admixture; Or-I, *O. rufipogon* I; Or-II, *O. rufipogon* II; Or-III, *O. rufipogon* III; ROOT, *O. meridionalis*. Scale bar, evolutionary distance of 6.0 x 10^-4^.


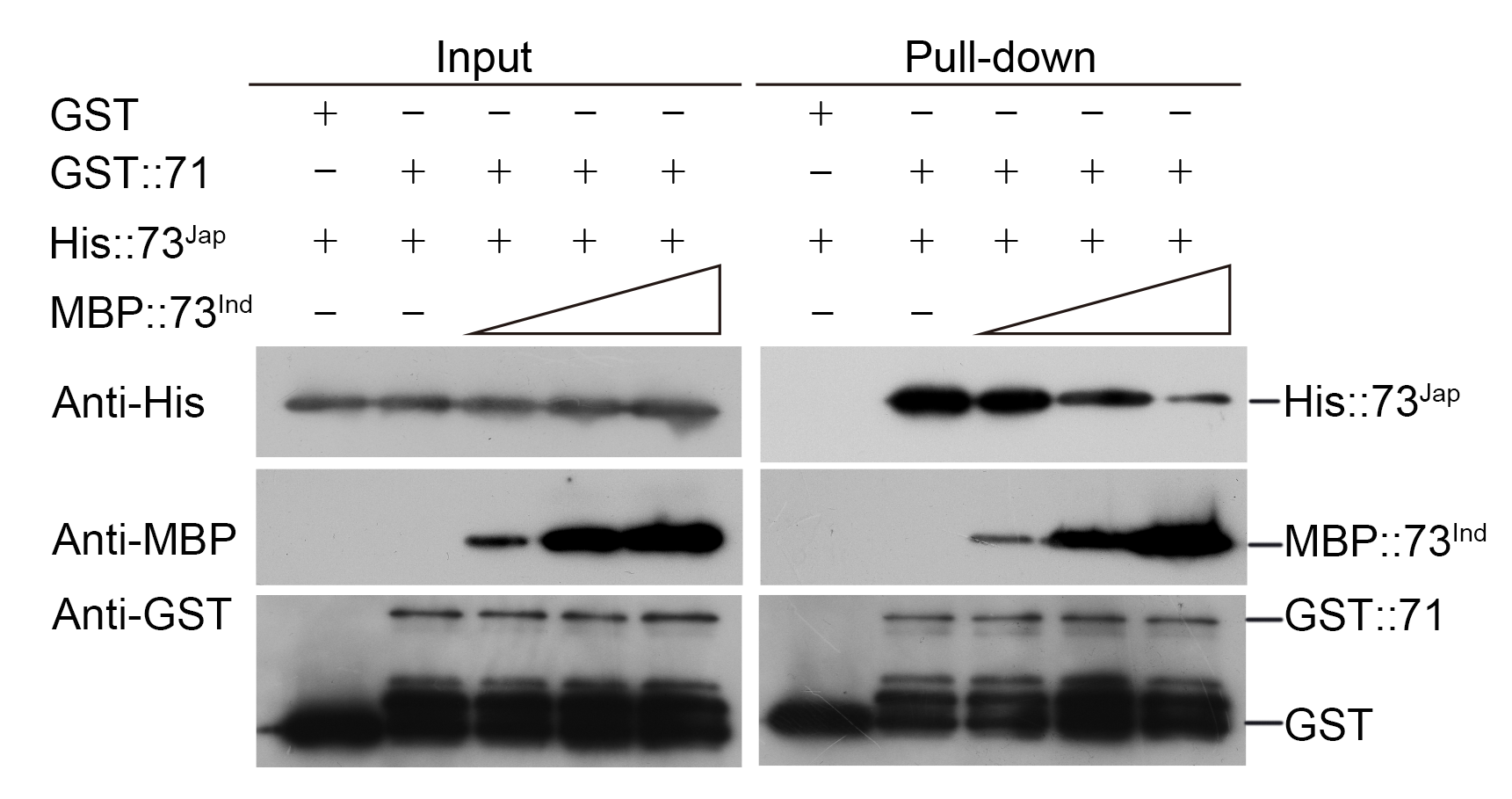


# **Supplementary Figure 12.** *In vitro* protein interaction between GST::bZIP71 and His ::bZIP73^Jap^ is weakened by the addition of MBP::bZIP73^Ind^. Left panel (Input), 10% of input before precipitation; Right panel (Pull-down), competitive pull-down using GST::bZIP71 as bait, while His::bZIP73^Jap^ or/and MBP::bZIP73^Ind^ as prey. Immunoprecipitated His::bZIP73^Jap^ and MBP::bZIP73^Ind^ were detected by the anti-His and anti-MBP antibodies, respectively. The gradient indicates increasing amount of MBP::bZIP73^Ind^.

**
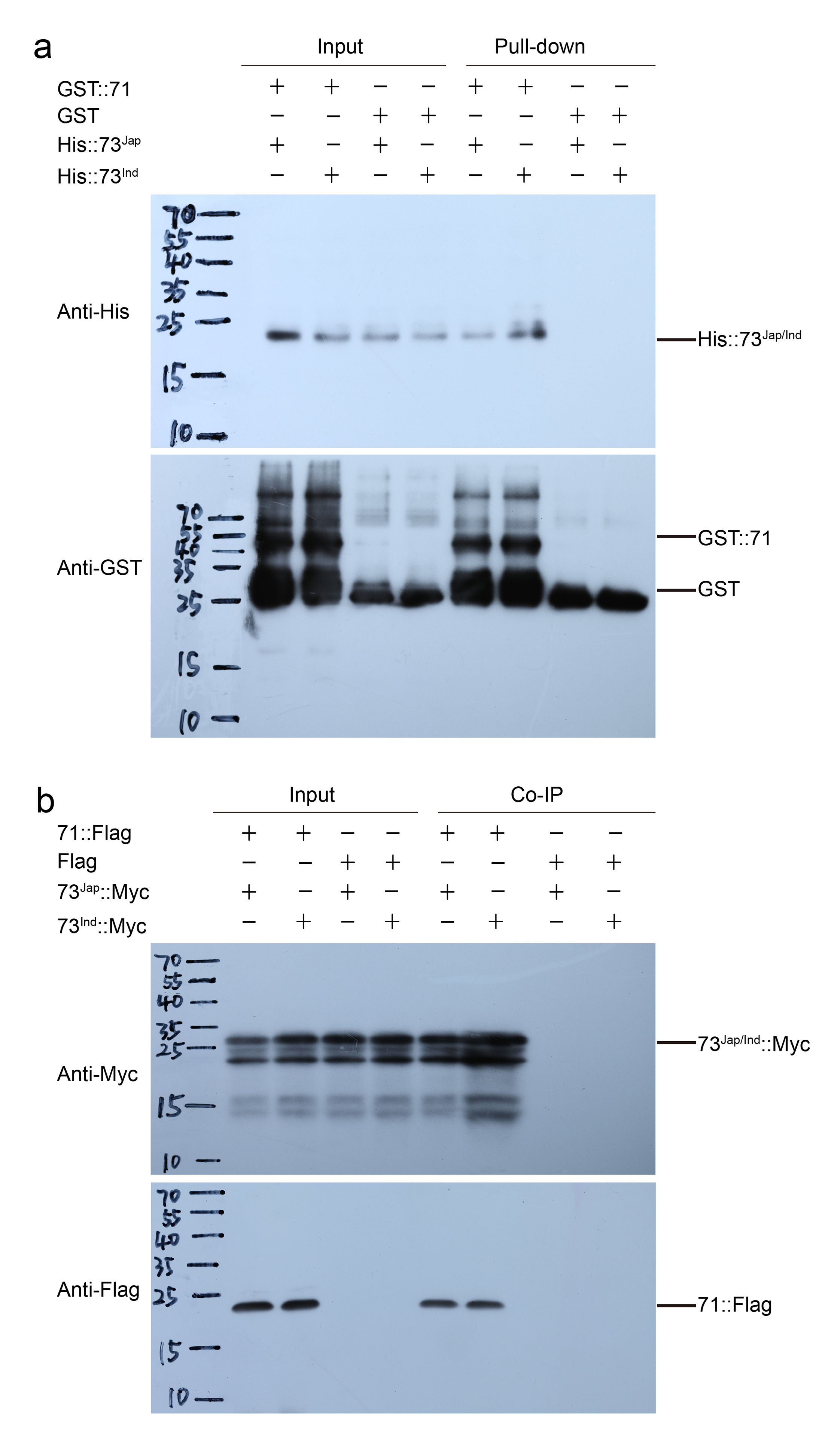
**

# **Supplementary Figure 13.** Original scans of the pull-down (**a**) and co-immunoprecipitation (**b**) blotting.

#
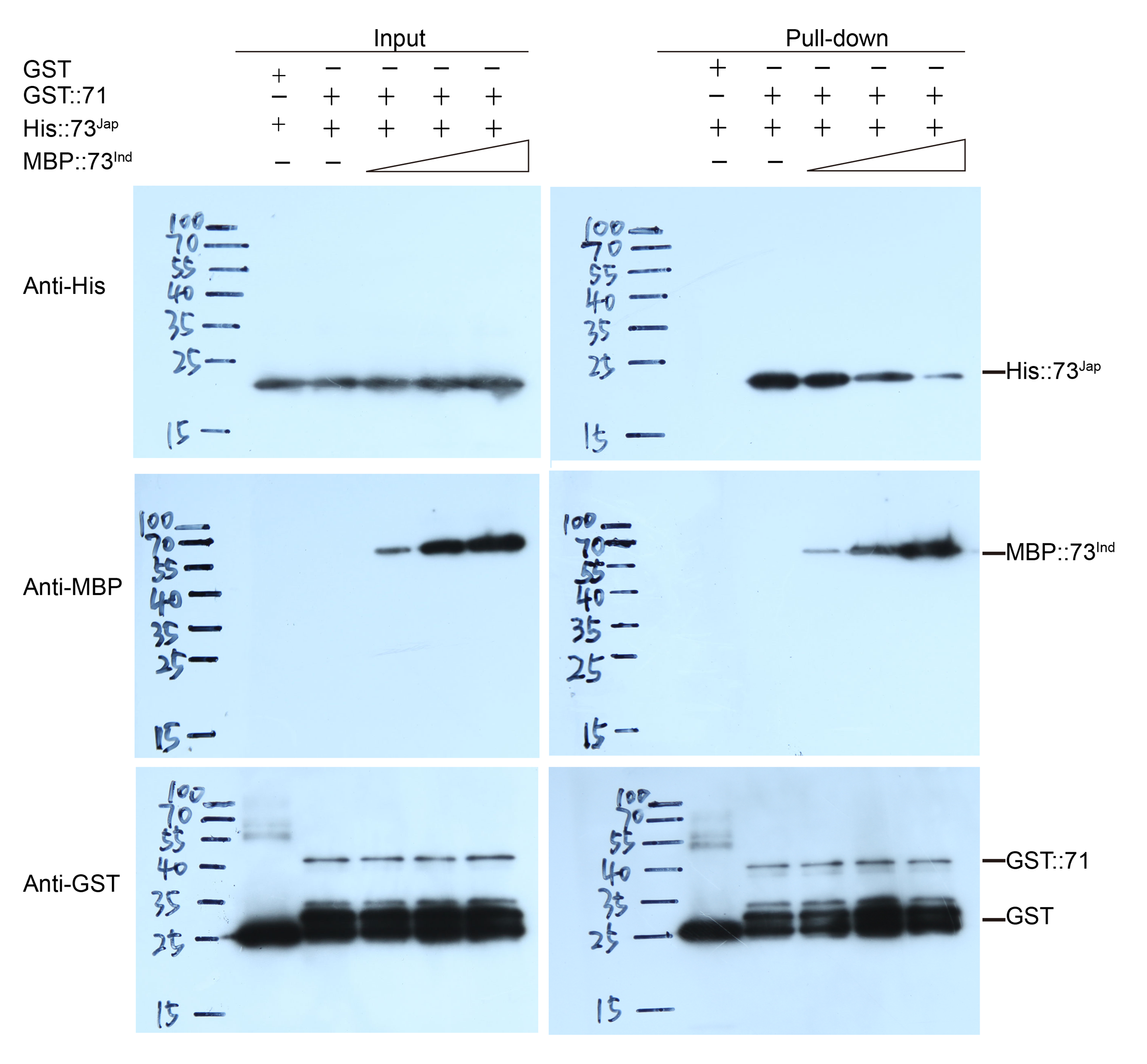


# **Supplementary Figure 14.** Original blotting scans of the competitive pull-down assay shown in **Supplementary Figure 12**.

# **Supplementary Table 1.** bZIP genes correlated with the low-temperature seedling survivability (LTSS) phenotype

| **LOC** | **Gene name** | **Chr** | **Position** | ***p**** | **Description** |
| --- | --- | --- | --- | --- | --- |
| *LOC_Os07g48820* | *OsbZIP63* (*rTGA2.1*; *OsNIF1*) | Chr7 | 29216374 | 0.002 | Plays a negative role in *Xanthomonas oryzae* defense^4^ |
| *LOC_Os01g59350* | *OsbZIP08* | Chr1 | 34310018 | 0.018 | bZIP domain-containing protein |
| *LOC_Os05g03860* | *OsbZIP38* (*LIP19*) | Chr5 | 1718443 | 0.033 | Dimerized with OsOBF1 and mediates low-temperature signal switching^5-8^ |
| *LOC_Os09g29820* | *OsbZIP73* | Chr9 | 18122850 | 0.040 | This study |
| *LOC_Os04g10260* | *OsbZIP35* | Chr4 | 5544994 | 0.040 | bZIP domain-containing protein |
| *LOC_Os09g28310* | *OsbZIP72* | Chr9 | 17190811 | 0.042 | Plays a positive role in drought resistance through ABA signaling^9^ |
| *LOC_Os06g10880* | *OsbZIP46* (*OsABF2*; *ABL1*) | Chr6 | 5677522 | 0.042 | Plays a positive role in drought, salinity, cold, oxidative stress, and ABA stress response through ABA signaling^10^ |
| *LOC_Os09g34880* | *OsbZIP76* | Chr9 | 20326768 | 0.044 | bZIP domain-containing protein |

*Association *p*-values were corrected by population covariance and adjusted using the FDR_BH method to control the false discovery rate in multiple testing.

# **Supplementary Table 2.** The list of bZIP genes with excessive LD and their transcription response to cold stress

| **Population*** | **MSU ID** | **Gene name** | **Seedling** | **Root** | **Reference** |
| --- | --- | --- | --- | --- | --- |
| Jap | *LOC_Os09g29820* | *OsbZIP73* | – | ↑ | – |
| Jap | *LOC_Os08g43090* | *OsbZIP68* | – | – | – |
| Ind | *LOC_Os01g58760* | *OsbZIP07* | – | – | – |
| Jap | *LOC_Os05g41540* | *OsbZIP44* | – | – | – |
| Jap | *LOC_Os03g58250* | *OsbZIP33/REB* | – | ↑ | ^11^ |
| Jap | *LOC_Os08g07970* | *OsbZIP64* | – | ↑ | – |
| Jap | *LOC_Os07g10890* | *OsbZIP59* | ↑ | – | – |
| Jap | *LOC_Os09g10840* | *OsbZIP70* | – | – | – |
| MC | *LOC_Os09g31390* | *OsbZIP74* | – | – | ^12^ |
| MC | *LOC_Os04g41820* | *OsbZIP36* | – | – | – |
| Ind | *LOC_Os01g64730* | *OsbZIP12* | ↑ | ↑ | ^13^ |
| MC, Jap, Ind | *LOC_Os12g09250* |  | – | ↑ | – |
| Jap | *LOC_Os02g52780* | *OsbZIP23* | ↑ | – | ^14^ |
| Jap | *LOC_Os02g10140* | *OsbZIP17* | – | ↑ | – |
| Ind | *LOC_Os02g03960* | *OsbZIP14* | ↑ | – | – |
| Jap | *LOC_Os02g10150* |  | – | ↑ | – |
| Ind | *LOC_Os03g20310* | *OsbZIP28* | ↑ | – | – |
| Jap, Ind | *LOC_Os03g21800* | *OsbZIP30/RF2b* | – | ↑ | ^15,16^ |
| Ind | *LOC_Os03g47200* | *OsbZIP31* | – | – | – |
| MC | *LOC_Os06g50310* | *OsbZIP53* | ↑ | – | – |
| Ind | *LOC_Os08g38020* | *OsbZIP67* | – | – | – |
| MC | *LOC_Os09g34060* | *OsbZIP75/RF2a* | – | – | ^15,16^ |
| Jap | *LOC_Os12g37410* | *OsbZIP87/OBF1* | ↑ | – | ^8^ |

*The occurrence of population with excessive LD. Jap, *japonica*; Ind, *indica*; MC, mini-core; ↑ up-regulated by cold treatment; – not responded to cold treatment.

# **Supplementary Table 3.** Allele frequency of the functional polymorphism (FNP) of the *bZIP73* gene in different rice ecotypes.

| **Population** | **A** | **G** | **N*** | **Sample size** | **Major Allele Frequency** |
| --- | --- | --- | --- | --- | --- |
| *O. sativa* ssp. *aus* | 24 | 3 | 9 | 36 | 0.89 |
| *O. sativa* ssp. *indica* | 49 | 4 | 13 | 66 | 0.92 |
| *O. sativa* ssp. *aromatic* | 6 | 1 | 1 | 8 | 0.86 |
| *O. sativa* ssp. *temperate japonica* | 0 | 23 | 7 | 30 | 1.00 |
| *O. sativa* ssp. *tropical japonica* | 0 | 21 | 6 | 27 | 1.00 |
| *O. rufipogon* I | 71 | 14 | 68 | 153 | 0.84 |
| *O. rufipogon* II | 6 | 82 | 27 | 115 | 0.93 |
| *O. rufipogon* III | 3 | 83 | 81 | 167 | 0.97 |

*Ambiguous due to low sequencing depth.

# **Supplementary Table 4.** Nucleotide diversity of *OsbZIP73* and its flanking regions in different rice subpopulations.

| **Population** | **Upstream** | ***bZIP73*** | **Downstream** |
| --- | --- | --- | --- |
| *O. sativa* ssp. *temperate japonica*^z^ | 0.00003A^u^ | 0.00005A | 0.00026A |
| *O. sativa* ssp. *tropical japonica*^y^ | 0.00002A | 0.00004A | 0.00012A |
| *O. sativa* ssp. *aromatic*^y^ | 0B | 0B | 0.00075A |
| *O. sativa* ssp. *aus*^x^ | 0.00000B | 0.00000B | 0.00002A |
| *O. sativa* ssp. indica^w^ | 0.00133A | 0.00016B | 0.00184A |
| *O. rufipogon* I^v^ | 0.00143AB | 0.00055B | 0.00227A |
| *O. rufipogon* II^v^ | 0.00208A | 0.00096A | 0.00239A |
| *O. rufipogon* III^v^ | 0.00184A | 0.00059B | 0.00173A |

^z^The Ryan-Einot-Gabriel-Welsch Q test (REGWQ) for non-significant ANOVA with the Satterthwaite approximation (Satterth) adjustment for unequal pool size

^y^REGWQ test with the Kenward-Roger (KR) adjustment for unequal variance

^x^REGWQ test with log transformation to improve unequal variances and the Satterth adjustment

^w^the Fisher's Least Significant Difference (LSD) test with KR adjustment

^v^LSD test with Satterth adjustment

^u^Means with different letters in a row are significantly different from each other (*α* = 0.05)

# **Supplementary References**

1. Wang, H. *et al.* The power of inbreeding: NGS-based GWAS of rice reveals convergent evolution during rice domestication. *Mol Plant* **9**, 975-985 (2016).

2. Huang, X. *et al.* A map of rice genome variation reveals the origin of cultivated rice. *Nature* **490**, 497-501 (2012).

3. Liu, L. *et al.* An integrative bioinformatics framework for genome-scale multiple level network reconstruction of rice. *J Integr Bioinform* **10**, 94-102 (2013).

4. Fitzgerald, H.A., Canlas, P.E., Chern, M.-S. & Ronald, P.C. Alteration of TGA factor activity in rice results in enhanced tolerance to *Xanthomonas oryzae* pv. *oryzae*. *Plant J* **43**, 335-347 (2005).

5. Aguan, K., Sugawara, K., Suzuki, N. & Kusano, T. Low-temperature-dependent expression of a rice gene encoding a protein with a leucine-zipper motif. *Mol Gen Genet* **240**, 1-8 (1993).

6. Shimizu, H., Berberich, T., Miyazaki, A., Imai, R. & Kusano, T. A heterodimer between LIP19 and OsOBF1 functions as a molecular switch in cold signaling in rice. *Plant Cell Physiol* **46**, S79-S79 (2005).

7. Shimizu, H., Thomas, B., Miyazaki, A., Imai, R. & Kusano, T. Significance of an interaction between LIP19 and OsOBF1 during cold stress in rice. *Plant Cell Physiol* **45**, S116-S116 (2004).

8. Shimizu, H. *et al.* LIP19, a basic region leucine zipper protein, is a fos-like molecular switch in the cold signaling of rice plants. *Plant Cell Physiol* **46**, 1623-1634 (2005).

9. Lu, G., Gao, C., Zheng, X. & Han, B. Identification of OsbZIP72 as a positive regulator of ABA response and drought tolerance in rice. *Planta* **229**, 605-615 (2009).

10. Hossain, M.A. *et al.* The ABRE-binding bZIP transcription factor OsABF2 is a positive regulator of abiotic stress and ABA signaling in rice. *J Plant Physiol* **167**, 1512-1520 (2010).

11. Yang, D., Wu, L., Hwang, Y.-S., Chen, L. & Huang, N. Expression of the REB transcriptional activator in rice grains improves the yield of recombinant proteins whose genes are controlled by a Reb-responsive promoter. *Proc Natl Acad Sci U S A* **98**, 11438-11443 (2001).

12. Lu, S.J. *et al.* Conservation of IRE1-regulated *bZIP74* mRNA unconventional splicing in rice (*Oryza sativa* L.) involved in ER stress responses. *Mol Plant* **5**, 504-514 (2012).

13. Joo, J., Lee, Y.H. & Song, S.I. Overexpression of the rice basic leucine zipper transcription factor OsbZIP12 confers drought tolerance to rice and makes seedlings hypersensitive to ABA. *Plant Biotechnol Rep* **8**, 431-441 (2014).

14. Dey, A., Samanta, M.K., Gayen, S., Sen, S.K. & Maiti, M.K. Enhanced gene expression rather than natural polymorphism in coding sequence of the *OsbZIP23* determines drought tolerance and yield improvement in rice genotypes. *PLOS One* **11**, 26 (2016).

15. Dai, S.H. *et al.* Transgenic rice plants that overexpress transcription factors RF2a and RF2b are tolerant to rice tungro virus replication and disease. *Proc Natl Acad Sci U S A* **105**, 21012-21016 (2008).

16. Liu, Y., Dai, S.H. & Beachy, R.N. Role of the C-terminal domains of rice (*Oryza sativa* L.) bZIP proteins RF2a and RF2b in regulating transcription. *Biochem J* **405**, 243-249 (2007).
