# Supplementary material for: Early selection of bZIP73 facilitated adaptation of japonica rice to cold climates
Source: Nat Commun. 2018 Aug 17;9:3302. doi: 10.1038/s41467-018-05753-w (PMC6098049; doi:10.1038/s41467-018-05753-w)
Supplement: Supplementary file 2 — Description of Additional Supplementary Files [file 41467_2018_5753_MOESM2_ESM.pdf]

Descriptions of Additional Supplementary Files

File Name: Supplementary Dataset 1
Description: Genes identified from the Y2H screening of the ZH11 cDNA library using BD::bZIP73Jap as bait.

File Name: Supplementary Dataset 2
Description: The 669 non-TE genes that were precipitated with bZIP73Jap::Flag.

File Name: Supplementary Dataset 3
Description: Distribution of G-boxes in promoters of OsNCED3, OsNCED5, and peroxidase precursor genes.

File Name: Supplementary Dataset 4
Description: The lowtemperature seedling survivability (LTSS) of the USDA mini-core population and the functional polymorphism (FNP) of the bZIP73 gene.

File Name: Supplementary Dataset 5
Description: Primers used in real time PCR analysis and construction of vectors.
